# Oncofetal dual‑specificity phosphatase 9 drives stem‐like properties through ERK1/2‐PPARG‐SCD axis‐mediated lipid metabolism in hepatocellular carcinoma

**DOI:** 10.1002/ctm2.70550

**Published:** 2025-12-12

**Authors:** Wang Peng, Hai Huang, Yuchong Zhao, Qiaodan Zhou, Mengdie Cao, Luyao Liu, Jingwen Liang, Haochen Cui, Shiru Chen, Wei Chen, Si Xiong, Bin Cheng, Shuya Bai

**Affiliations:** ^1^ Department of Gastroenterology and Hepatology Tongji Hospital Tongji Medical College Huazhong University of Science and Technology Wuhan China

**Keywords:** DUSP9, hepatocellular carcinoma, lipid metabolism, oncofetal reprogramming, prognostic signature, stemness

## Abstract

**Background:**

Oncofetal reprogramming—the reactivation of fetal‐like gene programmes in malignant cells—has been implicated in the progression and stemness of hepatocellular carcinoma (HCC), yet its protein landscape and connection to tumour stemness remain incompletely defined.

**Methods:**

We integrated multi‐omics datasets to derive an oncofetal reprogramming‐based prognostic signature (oncoScore) and validated it across multiple independent HCC cohorts. Candidate oncofetal proteins were validated in murine fetal liver and HCC tissue microarrays. Functional characterization of dual‐specificity phosphatase 9 (DUSP9) was performed using gain‐ and loss‐of‐function studies, including sphere and colony formation, proliferation, migration and invasion assays, sorafenib resistance testing, and limiting‐dilution tumourigenicity assays. Mechanistic studies employed Oil Red O staining, co‐immunoprecipitation, chromatin immunoprecipitation, pharmacologic inhibition and genetic rescue experiments.

**Results:**

The oncoScore demonstrated robust prognostic value across multiple independent HCC cohorts. DUSP9 emerged as a key regulator of stemness, promoting self‐renewal and aggressive phenotypes, enhancing sphere and colony formation, proliferation, migration, invasion, sorafenib resistance, and tumourigenicity. Mechanistically, DUSP9 drives lipid metabolism by upregulating stearoyl‐CoA desaturase (SCD) through the ERK1/2peroxisome proliferator‐activated receptor gamma (PPARG) signalling axis.

**Conclusion:**

Our results establish the oncoScore as a reliable prognostic marker for HCC and identify a DUSP‐9ERK1/2‐PPARG‐SCD pathway that links lipid metabolism to stemness. Targeting the oncofetal protein DUSP9 may offer a therapeutic avenue for aggressive, stemness‐driven HCC.

**Key points:**

- Oncofetal reprogramming‐based prognostic signature robustly stratifies HCC patient survival across independent cohorts.
- DUSP9 is identified as a core oncofetal regulator that drives stem‐like traits in HCC.
- Mechanistically, DUSP9 suppresses ERK1/2‐phosphorylation, stabilizes PPARG, and transcriptionally activates SCD.
- The DUSP9‐ERK1/2‐PPARG‐SCD axis remodels lipid metabolism to support proliferation, cell mobility, and sorafenib resistance.

## Graphical abstract

• A prognostic ‘oncoScore’ derived from multi‐omics and oncofetal reprogramming robustly stratifies HCC patient survival across independent cohorts.

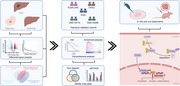

## 1 INTRODUCTION

Hepatocellular carcinoma (HCC) represents one of the most prevalent and lethal malignancies globally, characterized by a 5‐year survival rate of approximately 18% due to elevated relapse frequency, metastatic capability and therapeutic resistance.^1^, ^2^ Accumulated evidence highlights the pivotal roles of cancer stem cells (CSCs) in driving treatment resistance and tumour progression, owing to their capacities of self‐renewal, unlimited proliferation, multilineage differentiation and resistance to chemotherapy.^3^, ^4^ Therefore, deciphering the mechanisms that govern CSC properties proves crucial for improving therapeutic approaches and patient prognosis.

Oncofetal reprogramming refers to the pathological process wherein cancer cells reacquire fetal‐like characteristics, recapitulating embryonic developmental traits such as hyperproliferative capacity, cellular plasticity and elevated expression of oncofetal proteins.^5^, ^6^ Oncofetal proteins, typically highly expressed during embryogenesis but silenced or minimally expressed in healthy adult tissues, are aberrantly re‐expressed in malignancies, making them promising diagnostic biomarkers and therapeutic targets.^7^, ^8^ The reactivation of oncofetal genes suggests that they could serve pivotal functions in cancer initiation and progression. Given the phenotypic parallels between CSCs and embryonic stem cells, oncofetal proteins may serve as critical regulators of tumour stemness.^9^, ^10^ Despite their recognized importance, the full landscape of oncofetal proteins in HCC and their connection to tumour stemness remains to be systematically explored.

Metabolic reprogramming represents a distinctive feature of malignancy. Metabolic enzymes coordinate complex networks to meet heightened bioenergetic and biosynthetic demands.^11^ Lipid metabolism, in particular, has been increasingly recognized as a key regulator of CSC properties.^12^ Fatty acid metabolism governs lipid homeostasis through coordinated anabolic and catabolic processes, supporting membrane biogenesis, signal transduction and energy production.^13^ Stearoyl‐CoA desaturase (SCD), a critical enzyme controlling fatty acid desaturation, catalyses the production of lipids essential for maintaining membrane fluidity and cellular signalling, and aberrant SCD activity has been implicated in promoting CSC properties in various cancers.^14^, ^15^, ^16^, ^17^

This study investigated the landscape of oncofetal reprogramming in HCC using an integrative multi‐omics approach. Initially, we identified 23 key oncofetal genes exhibiting elevated expression not only in HCC but also across multiple cancer types. Using this oncofetal gene set, we constructed an oncofetal reprogramming‐based prognostic signature (oncoScore) in HCC patients. Functional enrichment analysis of the oncoScore further underscored the connection among oncofetal reprogramming, tumour stemness and tumour aggressiveness. We next identified dual specificity phosphatase 9 (DUSP9) as the key oncofetal protein driving HCC stemness and poor prognosis. Mechanistically, DUSP9 sustained peroxisome proliferator‐activated receptor gamma (PPARG) stability via ERK1/2 dephosphorylation, leading to PPARG‐mediated transcriptional upregulation of SCD. This DUSP9–ERK1/2–PPARG–SCD axis promoted lipid metabolism to support CSC properties in HCC. These findings underscore DUSP9's potential as a therapeutic target for HCC.

## 2 MATERIALS AND METHODS

More detailed experimental procedures and reagent information are provided in the Supporting Information section.

### 2.1 Data collection and bioinformatics analysis

Publicly available omics datasets were obtained from the Gene Expression Omnibus (GEO), Cancer Dependency Map (DepMap), LIver cancer MOdel REpository (LIMORE), Clinical Proteomic Tumour Analysis Consortium (CPTAC), HCCDB and The Cancer Genome Atlas (TCGA) databases. Detailed data types and sources are listed in Table S1. For survival analyses, only cases with overall survival (OS) above 30 days were included to avoid non‐malignancy‐associated mortality. Cellular differentiation potential was estimated using CytoTRACE (https://cytotrace.stanford.edu/), enabling pseudotime trajectory analysis of single‐cell RNA sequencing (scRNA‐seq) data and assessment of the relationship between *DUSP9* expression and differentiation status in poorly differentiated hepatoblast.^18^ The ‘single‐cell flux estimation analysis (scFEA)’ Python package was used to infer metabolite abundance from scRNA‐seq data.^19^ Gene Ontology (GO), Kyoto Encyclopaedia of Genes and Genomes (KEGG) pathway enrichment analyses and gene set enrichment analysis (GSEA) were performed via the ‘clusterProfiler’ R package.^20^ Kaplan–Meier survival analysis was conducted with the ‘survminer’ R package. The restricted mean survival time (RMST) was calculated by the ‘survRM2’ R package. The oncofetal programming‐based signature was established by calculating the oncofetal score (oncoScore) via the following formula:

$$\mathrm{OncoScore}=\sum_{i=1}^{n}\mathrm{Coef}\left(\mathrm{gen}e_{i}\right)\times \mathrm{Expression}\left(\mathrm{gen}e_{i}\right)$$

The oncoScore coefficients were estimated using a standard Cox proportional hazards model (no LASSO penalty).

### 2.2 Clinical liver samples

Paired tumour and adjacent liver tissues were procured from patients at Tongji Hospital, Tongji Medical College, Huazhong University of Science and Technology (HUST), Wuhan, China. All patients were pathologically confirmed with primary HCC and had not received any preoperative treatments. The experimental protocol was authorized by the Ethics Committee of Tongji Hospital (TJ‐IRB20230234), and informed consent was acquired from all subjects.

### 2.3 Cell lines and cell culture

The L02 normal hepatic cell line and HCC cell lines (MHCC‐97L, MHCC‐97H, MHCC‐LM3) were sourced from the Cell Bank of the Chinese Academy of Sciences. HLE and HLF cells were acquired from JCRB Cell Bank, whereas SNU‐398, HepG2, Huh7, PLC/PRF/5, HEK‐293T and Hepa1‐6 were purchased from ATCC. Cell culture was performed in DMEM containing 10% fetal bovine serum (FBS, GIBCO) at 37°C with 5% CO_2_. For chemical treatments, the ERK1/2 inhibitor SCH772984 (MedChemExpress, HY‐50846) and/or proteasome inhibitor MG132 (MedChemExpress, HY‐13259) were applied for a 24‐h incubation.

### 2.4 Mouse fetal liver isolation

C57BL/6 mice (4 weeks) were purchased from GemPharmatech and maintained under SPF conditions. The mature male and the female mice were housed together overnight at a 1:2 ratio. The next morning, female mice were monitored for vaginal plugs. Those that were plug‐positive were then single‐housed and designated as being at embryonic day .5 (E.5). At E13.5, pregnant mice were euthanized, and embryos were dissected from the uterus. Fetal liver tissues were carefully isolated using ophthalmic forceps. Maternal liver tissues were also collected. All tissues were stored at −80°C for subsequent experiments. All animal experiments were performed in compliance with the ethical guidelines approved by the Institutional Review Board of Tongji Hospital (TJH‐202212058).

### 2.5 Quantitative real‐time polymerase chain reaction (qRT‐PCR)

Total RNA was extracted from samples using Trizol reagent (Invitrogen), and cDNA was subsequently generated with HiScript III Reverse Transcriptase (Vazyme, R302‐01). For quantitative real‐time polymerase chain reaction (qRT‐PCR), the ChamQ Universal SYBR qPCR Master Mix (Vazyme, Q711‐02) was utilized. Data analysis involved the 2^−ΔΔ^ *^Ct^* (Ct, cycle threshold) method to ascertain relative gene expression, and the primer sequences can be found in Table S2.

### 2.6 Western blot

Western blotting was conducted following an established protocol.^21^ The primary antibodies employed in this study are listed as follows: α‐tubulin (Proteintech, 66031‐1‐Ig), DUSP9 (Proteintech, 26718‐1‐AP), OCT4 (Proteintech, 11263‐1‐AP), NANOG (Proteintech, 14295‐1‐AP), SOX2 (ABclonal, A11501), SCD (Proteintech, 28678‐1‐AP), PPARG (Proteintech, 16643‐1‐AP), H3 (Huabio, EM30605), ERK1/2 (ABclonal, A4782), p‐ERK1/2 (ABclonal, AP0974), P38 (Huabio, ET1702‐65), Phospho‐p38 (Huabio, HA722669), JNK1/2/3 (Huabio, ET1601‐28) and p‐JNK1/2/3 (Huabio, ET1609‐42).

### 2.7 Plasmid construction, lentivirus packaging and transduction

The *DUSP9* (NM_001395.4) overexpression plasmid (and its control) and the *DUSP9* knockdown plasmid (and its control) were procured from Genomeditech and GeneChem, respectively. The lentivirus packaging plasmids psPAX2 and pMD2.G, as well as the lentivirus interference vector plasmid pLKO.1‐puro, were acquired from Addgene and maintained by our laboratory. The *SCD* knockdown plasmid was constructed in‐house using the pLKO.1‐puro backbone, whereas the *SCD* overexpression plasmid was generated using the empty vector from the *DUSP9* overexpression system. The primers used for plasmid construction can be found in Table S3.

Lentiviral particles were produced using a three‐plasmid packaging system. HEK‐293T cells were co‐transfected with the target plasmid or control plasmid and two packaging plasmids at a ratio of 2:1:1. The supernatant was collected at 48 and 72 h, centrifuged and filtered (.45 µm) and stored for subsequent cell infection. As for lentivirus transduction, target cells were incubated with lentiviral supernatant supplemented with 5–10 µg/mL polybrene. After 72 h, use complete medium containing puromycin or hygromycin to establish stable cell lines.

### 2.8 Sphere formation assay

Cells were seeded in 24‐well low‐attachment plates (Corning) at a density of 2000 cells per well. They were cultured in tumour sphere medium, which consisted of DMEM/F12 supplemented with 20 ng/mL EGF (GIBCO, PHG0311), 10 ng/mL bFGF (GIBCO, PHG0266), 2% B27 (GIBCO, 17504‐044), 1% N2 (GIBCO, 17502‐048), 1% methyl cellulose (Sigma‐Aldrich, M0262) and 1% penicillin/streptomycin. The cultures were replenished with 100 µL of fresh medium every 3 days. After 10–14 days, the resulting spheres were imaged under a microscope.

### 2.9 CCK8 proliferation and sorafenib resistance assay

For CCK8 proliferation assays, cells were seeded in 96‐well plates at 1000 cells/well. Four identical plates were prepared for parallel measurement at 0, 24, 48 and 72 h. At each time point, the medium was replaced with 100 µL of a mixture containing 90 µL serum‐free DMEM and 10 µL CCK8 reagent. After 1 h of incubation at 37°C in the dark, the absorbance at 450 nm was recorded using a microplate reader (Thermo Fisher). For sorafenib sensitivity, 3000 cells/well were treated with varying sorafenib concentrations (MedChemExpress, HY‐10201; 0, 5, 10, 15, 20 µM) for 24 h post‐adhesion, after which absorbance at 450 nm was measured as described.

### 2.10 Long‐term colony formation assay

HCC cells were plated in six‐well plates at 2500 cells/well. After 24 h of attachment, cells were exposed to either DMSO (control) or 20 µM sorafenib for 10–14 days. The resulting colonies were fixed with 4% paraformaldehyde, stained with .1% crystal violet and quantified using ImageJ software.

### 2.11 In vivo limiting dilution tumourigenicity assay

Male BALB/c nude mice (4 weeks old) procured from GemPharmatech were housed under specific pathogen‐free conditions. Cells were subcutaneously inoculated at varying cell concentrations (5 × 10^5^, 5 × 10^4^ and 5 × 10^3^ cells per mouse; *n* = 4 each group). Cells were trypsinized, centrifuged and resuspended in PBS. Cell concentrations were adjusted and kept on ice until injection. The matrix gel and cell suspension were mixed at 1:1 ratio and subcutaneously injected into the back of the right hind limb of the mouse. The tumour volume was monitored every 72 h by calliper measurements and determined by the formula (length × width × width)/2. Once the experimental period reached around 30 days or if humane endpoints were met, the mice were sacrificed. The ensuing tumours were then collected and imaged for documentation. All procedures involving animals were performed in accordance with the ethical standards approved by the Tongji Hospital Institutional Review Board (TJH‐202212058).

### 2.12 ALDH activity assay

ALDH activity was assessed with a commercial assay kit (Abbkine, KTB3032) per the manufacturer's instructions. In brief, NADH standards and prepared cell samples were transferred to a 96‐well plate. A 200 µL reaction mixture containing acetaldehyde and the ALDH substrate was then added to each well. After a 1‐h incubation at room temperature, the absorbance at 340 nm was recorded using a microplate reader.

### 2.13 Fatty acid oxidation (FAO) activity assay

Fatty acid oxidation (FAO) activity was evaluated using a colorimetric assay kit (Elabscience, E‐BC‐K784‐M) according to the manufacturer's instructions. Cell homogenates were centrifuged at 10 000 *g* for 10 min at 4°C to collect supernatants. Following protein concentration normalization, absorbance was measured at 450 nm, and FAO activity was calculated as previously described.^22^

### 2.14 Co‐immunoprecipitation (Co‐IP) assay

Cell lysates prepared with NP‐40 lysis buffer were incubated overnight at 4°C with the target antibody. Protein A/G magnetic beads (MCE, HY‐K0202) were then added for a 4‐h incubation. After extensive washing, bound proteins were eluted by boiling and analysed by Western blot using an ExactBlot secondary antibody (Zenbio, #550124). An aliquot of the lysate was reserved as the input control.

### 2.15 Chromatin immunoprecipitation (ChIP)

Chromatin immunoprecipitation (ChIP) assays were performed with a commercial kit (BIOLOGY, BOLG2309). After cross‐linking cells with 1% formaldehyde and lysing, chromatin was sheared by sonication to 100–500 bp fragments. Immunoprecipitation was carried out using an anti‐PPARG antibody (Proteintech, 16643‐1‐AP) or a control IgG (Proteintech, 30000‐0‐AP). Precipitated DNA was quantified by qPCR with specific primers (Forward: GCTCCTACAGACACGGAAAAG; Reverse: GCTCAGGACCATATTGCCCTC).

### 2.16 Statistical methods

All in vitro experiments included three independent biological replicates (*n* = 3) unless otherwise stated. The results are presented as the mean ± standard deviation (SD). Statistical analyses and data visualizations were performed in GraphPad Prism 8.4.2 and R 4.0.2. Two‐tailed tests were applied universally. The appropriateness of distribution determined the use of Student's *t*‐test or Mann–Whitney–Wilcoxon test for continuous variables. Spearman's method evaluated correlations, whereas chi‐square and Fisher's exact tests analysed categorical data associations. Survival differences were assessed with the log‐rank test. Significance levels are denoted in figures as follows: ns (not significant), *p* > .05; *p < .05; **p < .01; ***p < .001; ****p < .0001.

## 3 RESULTS

### 3.1 Construction and validation of an oncofetal reprogramming‐based prognostic signature in HCC patients across five public datasets

**FIGURE 1:**
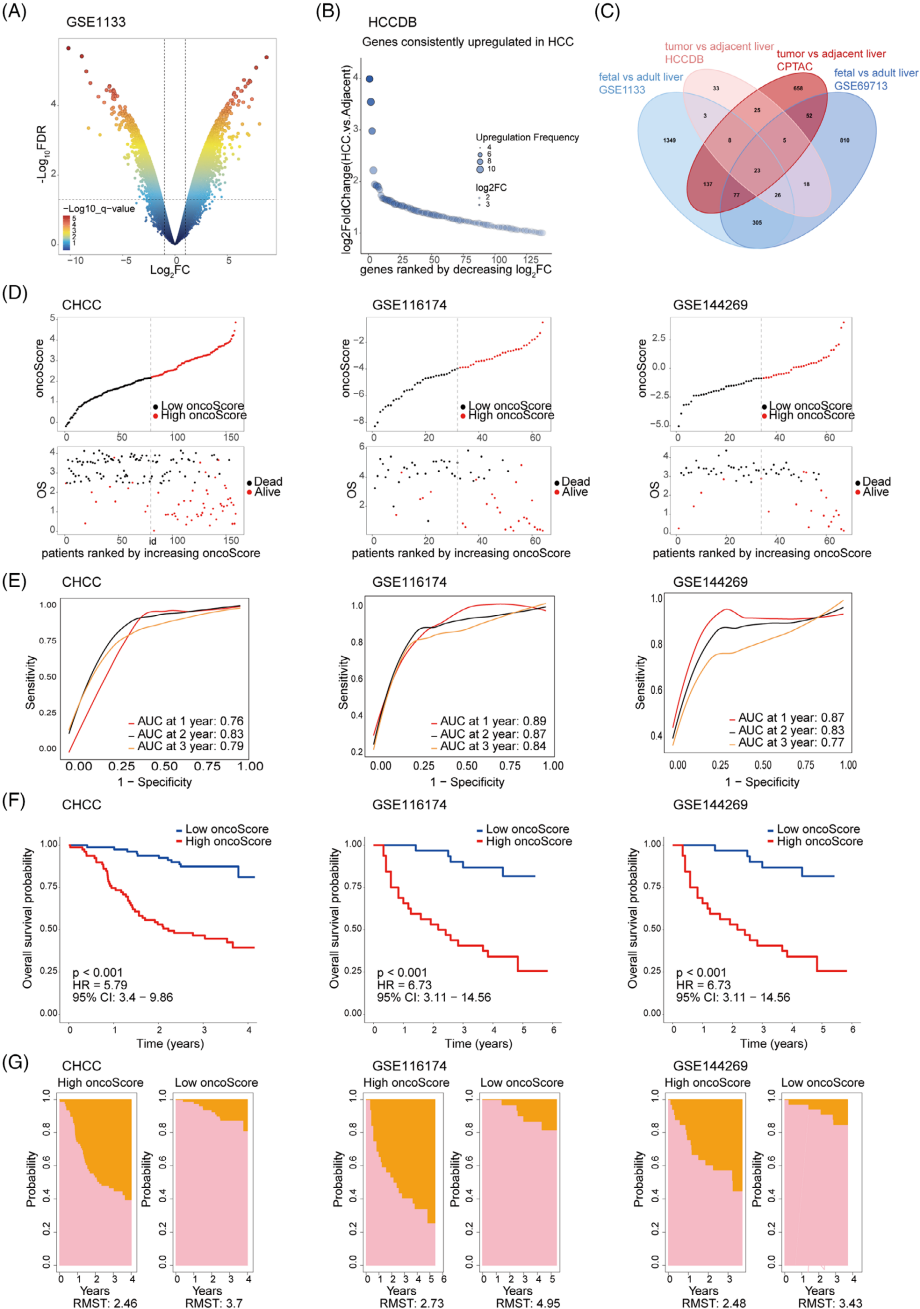
Construction and validation of oncofetal reprogramming‐based prognostic signature in hepatocellular carcinoma (HCC) patients across five public datasets. (A) Volcano plot showing differentially expressed genes in the GSE1133 dataset (fetal vs. adult liver). (B) Rank plot showing genes consistently upregulated in HCC in HCCDB database. (C) Venn diagram showing the intersection of genes/proteins elevated in both fetal livers and HCC tumours. (D) Distribution of the oncoScore signature and overall survival status of HCC patients in the CHCC, GSE116174 and GSE144269 datasets. (E) Time‐dependent receiver operating characteristic (ROC) curves for 1–3‐year OS across HCC datasets. (F) Kaplan–Meier survival analysis of OS for patients with high and low oncoScore across HCC datasets. (G) Restricted mean survival time between patients with high and low oncoScore across HCC datasets. Survival analysis was performed using the log‐rank test. AUC, area under the receiver operating characteristic curve; CI, confidence interval; HR, hazard ratio; RMST, restricted mean survival time.

We aimed to systematically identify genes with robust oncofetal expression characteristics in HCC and evaluate their prognostic values. To enhance the robustness of gene selection, we applied a gene‐overlapping approach based on the oncofetal reprogramming concept using different datasets. Genes with higher expression in fetal compared to adult liver were derived from the GSE1133 and GSE69713 datasets (fold change > 2, *p* < .05), and genes with higher expression in HCC compared to adjacent tissues were derived from the CPTAC proteomics (fold change > 1.414, *p* < .05) and the HCCDB transcriptomic database (fold change > 2, *p* < .05) (Figure 1A,B; Figure S1A,B). Through intersection of differentially upregulated genes from these analyses, we obtained 23 genes with robust oncofetal expression characteristics in HCC. Importantly, our gene selection process was independent of prognosis, thereby eliminating circularity risk when validating the prognostic value of these oncofetal genes (Figure 1C; Table S4). Further pan‐cancer analysis revealed that these 23 genes were not only upregulated in HCC but also displayed elevated expression in most other cancer types compared to adjacent non‐tumour or normal tissues (Figure S1C). This indicates that these oncofetal genes represent a broader oncofetal reprogramming signature associated with tumourigenesis.

To further explore the cellular expression of these oncofetal genes in HCC, we utilized scRNA‐seq data from the GSE16635 dataset. Cells were annotated into four major types based on canonical markers: endothelial cells, epithelial cells, fibroblasts and immune cells (Figure S1D,E). Analysis of the expression patterns of the 23 oncofetal genes across these cell types revealed that 21 out of 23 genes were predominantly expressed in epithelial cells (Figure S1F), which are the primary malignant component of HCC. Specifically, *MCM2* showed higher expression in immune cells and fibroblasts, whereas *ROBO1* was primarily expressed in endothelial cells (Figure S1G). These findings suggest that the majority of oncofetal genes identified in HCC are closely associated with the malignant epithelial compartment, which aligns with the fact that HCC is an epithelial‐derived carcinoma.

Using these 23 genes, we constructed a prognostic signature (oncoScore) via Cox proportional hazards regression. The oncoScore was calculated for each patient in five public datasets (TCGA‐LIHC, CHCC, ICGC‐LIRI, GSE116174 and GSE144269), and patients were stratified into high‐ and low‐oncoScore groups based on the median value (Figure 1D; Figure S2A). The predictive performance of the oncoScore was assessed using time‐dependent receiver operating characteristic (ROC) curves, Kaplan–Meier (K–M) survival analysis and RMST analysis. The ROC curves demonstrated robust predictive capability, with area under the curve (AUC) values ranging from .73 to .89 across the five datasets (Figure 1E; Figure S2B). K–M analysis revealed that patients with high oncoScore exhibited significantly worse OS compared to the low oncoScore group (Figure 1F; Figure S2C). Consistently, RMST analysis showed that high oncoScore was associated with reduced survival time (Figure 1G; Figure S2D).

These results collectively demonstrate that the oncoScore is a reliable and consistent predictor of OS in HCC patients. The fact that 21 of the 23 oncofetal genes are predominantly expressed in epithelial cells, which constitute the primary malignant population in HCC, further indicates that the oncoScore primarily captures the oncofetal reprogramming characteristics of malignant epithelial cells. This highlights its strong association with tumour aggressiveness.

### 3.2 The oncoScore signature predicts poor prognosis in HCC patients

We next assessed the independent prognostic value of the oncoScore using univariate and multivariate Cox regression analyses in four public HCC datasets. The oncoScore was consistently identified as an independent predictor of OS, with adjusted hazard ratios (HRs) of 3.36 (95% confidence interval [CI]: 1.77–6.40) in TCGA‐LIHC (Figure S3A), 4.54 (95% CI: 2.28–9.04) in CHCC (Figure S3B), 3.15 (95% CI: 1.67–5.94) in ICGC‐LIRI (Figure S3C) and 7.41 (95% CI: 2.50–21.95) in the GSE116174 dataset (Figure S3D). Only significant variables (*p* < .05) in the univariable Cox analyses were included into multivariable Cox analyses. Across all four cohorts, oncoScore remained independently prognostic and consistently showed higher HRs than clinical staging covariates, including BCLC and/or TNM staging. However, established clinical staging systems failed to reach significance across all datasets. In CHCC dataset, BCLC and TNM staging were not significant in the multivariable model. In GSE116174 dataset, clinical staging failed to reach significance in univariate analysis (*p* = .295) and was therefore excluded from multivariate modelling, whereas oncoScore maintained strong prognostic significance (HR = 7.41, 95% CI: 2.50–21.95, *p* = .001). Furthermore, we explored the association between oncoScore and key clinicopathologic features across these datasets. In the TCGA‐LIHC dataset, a high oncoScore was significantly associated with elevated serum alpha‐fetoprotein (AFP) levels and advanced tumour grade. In the CHCC dataset, oncoScore was correlated with higher serum AFP levels, presence of tumour thrombus and advanced BCLC stage. In GSE116174, it was associated with increased tumour invasion (Figure S3E). The C‐index plots also showed that oncoScore had relatively higher C‐index values and narrower 95% CIs than clinical staging systems such as BCLC/TNM staging (Figure S4A). We also compared prognostic performance of oncoScore with reported oncofetal biomarker panels, and oncoScore showed improved discrimination, as reflected by higher C‐index values (Figure S4B). These findings indicate that beyond being an independent prognostic marker, the oncoScore also reflects aggressive clinicopathologic characteristics, further underscoring its clinical relevance.

### 3.3 Functional annotation of the oncoScore signature in the TCGA‐LIHC dataset

**FIGURE 2:**
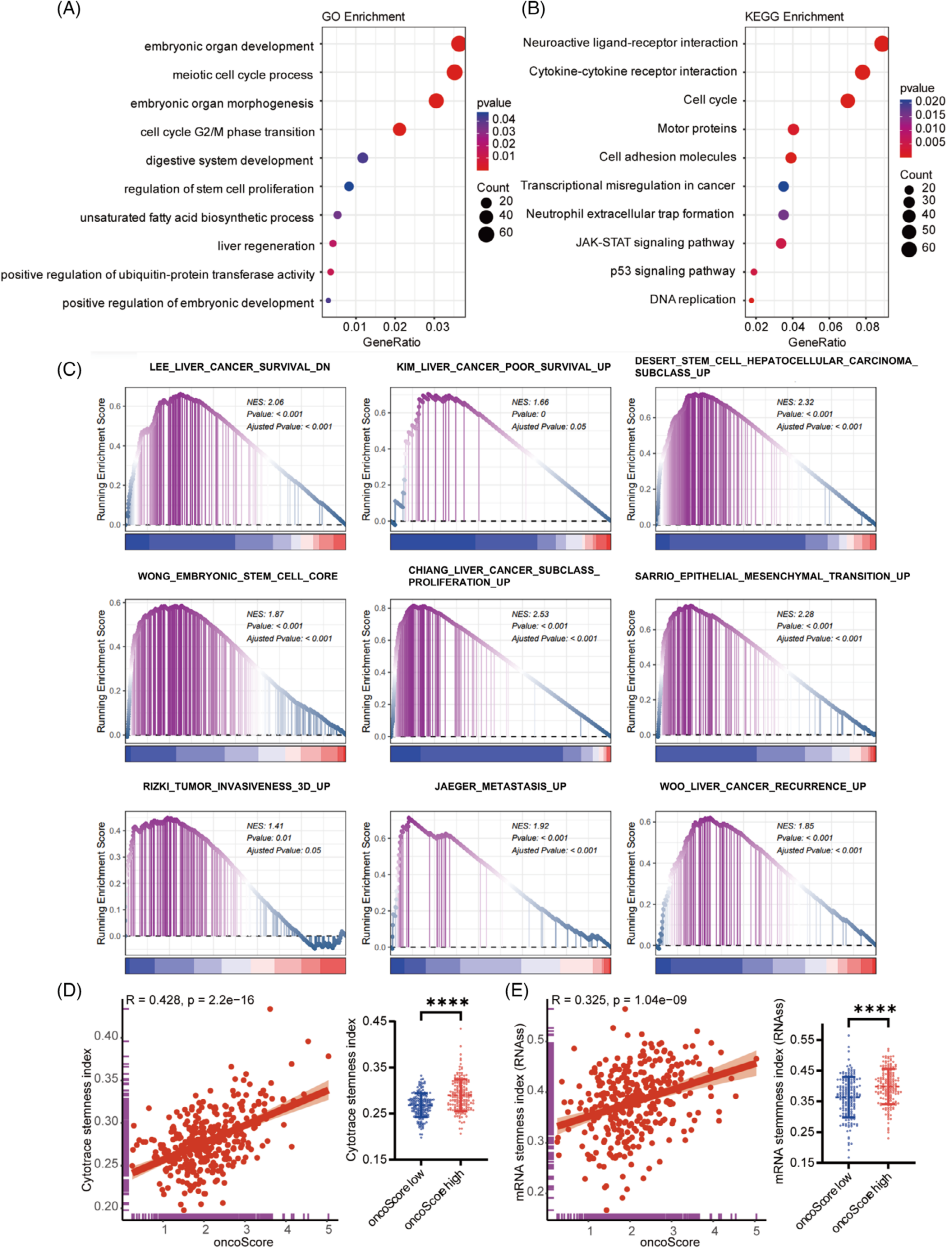
Functional annotation of the oncoScore signature in the TCGA‐LIHC dataset. (A) Gene Ontology (GO) functional enrichment analysis of the oncoScore. (B) KEGG functional enrichment analysis of the oncoScore. (C) GSEA analysis of the oncoScore (D) Scatter plot and beeswarm plot showing the correlation between the oncoScore levels and the Cytotrace‐based stemness index. (E) Scatter plot and beeswarm plot showing the correlation between the oncoScore levels and the mRNA‐based stemness index. Data are presented as mean ± SD and analysed using Student's *t*‐test. Spearman's rank correlation test was used to analyse the correlation between continuous variables. ****p < .0001. NES, normalized enrichment score.

To elucidate the potential functional roles of the oncoScore signature in HCC, we first performed GO and KEGG enrichment analyses in the TCGA‐LIHC dataset. The results showed significant enrichment of biological processes associated with embryonic organ development, regulation of stem cell proliferation and liver regeneration (Figure 2A), consistent with the oncofetal reprogramming nature of the oncoScore. In addition, KEGG pathway analysis indicated that the oncoScore was closely associated with pathways related to cell cycle, motor proteins and transcriptional misregulation in cancer (Figure 2B), suggesting that the oncoScore also reflects characteristics of tumour aggressiveness.

To further explore the biological relevance of the oncoScore in HCC, we conducted GSEA, which demonstrated that the high‐oncoScore group was significantly enriched in pathways indicative of poor prognosis and aggressive tumour behaviour. These pathways included ‘KIM‐Liver Cancer Poor Survival UP’, ‘DESERT‐Stem Cell HCC Subclass Up’, ‘WONG‐Embryonic Stem Cell Core’, ‘CHIANG‐Liver Cancer Subclass Proliferation UP’, ‘SARRIO‐Epithelial Mesenchymal Transition UP’, ‘RIZKI‐Tumour Invasiveness 3D UP’, ‘JAEGER‐Metastasis UP’ and ‘WOO‐Liver Cancer Recurrence UP’ (Figure 2C). These results strongly suggest that the oncoScore is not only linked to oncofetal reprogramming but also to HCC stemness, a key driver of tumour malignancy.

Given the close relationship between oncofetal reprogramming and tumour stemness, we further investigated the association between the oncoScore and two widely recognized stemness indices in HCC. Specifically, we evaluated the correlation between the oncoScore and two previously established stemness index, Cytotrace‐based stemness index^18^ (Figure 2D) and mRNA‐based stemness index^23^ (Figure 2E). Both analyses revealed a significant positive correlation, indicating that patients with higher oncoScore exhibited markedly elevated stemness indices (*p* < .0001). This strong positive association further supports that the oncoScore reflects stemness features in HCC.

Collectively, these findings demonstrate that the oncoScore signature captures key characteristics of oncofetal reprogramming and is strongly associated with stemness properties in HCC. This provides a foundation for further exploration of the oncoScore's role in promoting HCC stemness and malignant behaviour.

### 3.4 Multi‐omics integration uncovers DUSP9 as a key oncofetal protein in hepatocellular carcinoma

**FIGURE 3:**
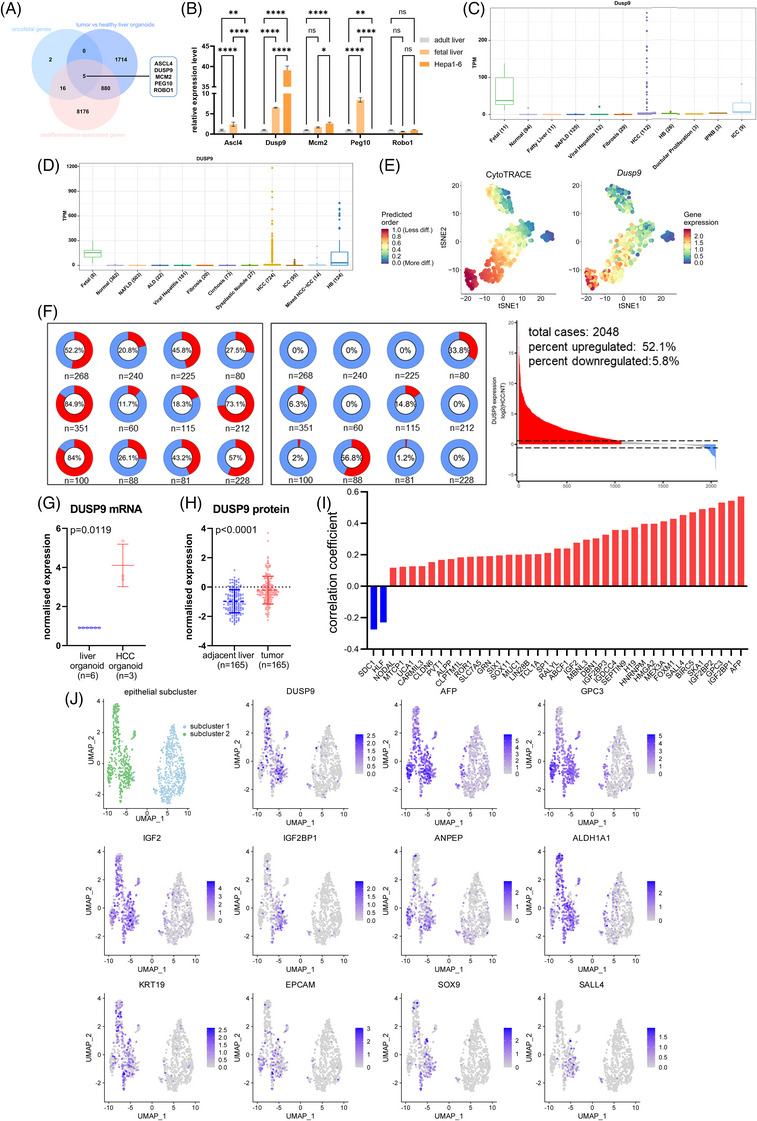
Multi‐omics integration uncovers dual‑specificity phosphatase 9 (DUSP9) as a key oncofetal protein in hepatocellular carcinoma. (A) Venn diagram showing the five overlapping genes among oncofetal genes, dedifferentiation‐associated genes and genes upregulated in hepatocellular carcinoma (HCC)‐derived organoids (compared to healthy liver organoids). (B) mRNA expression of the five genes in murine adult liver, fetal liver and Hepa1‐6 cells. (C) Murine *Dusp9* mRNA expression in fetal, normal and diseased liver tissues in the GepLiver database. (D) Human *DUSP9* mRNA expression in fetal, normal and diseased liver tissues in the GepLiver database. (E) CytoTRACE pseudotime analysis of hepatoblast single‐cell sequencing data. Left: cell differentiation states (red indicates lower differentiation). Right: *DUSP9* expression (red indicates higher expression). (F) Expression pattern of *DUSP9* across 12 HCC bulk transcriptomic datasets. Left: proportion of *DUSP9* upregulation. Centre: proportion of downregulation. Right: overall distribution in 2048 HCC patients. (G) *DUSP9* mRNA expression in HCC‐derived organoids (*n* = 3) versus healthy liver organoids (*n* = 6). (H) DUSP9 protein expression in paired HCC and adjacent tissues (*n* = 165 pairs) from the CPTAC dataset. (I) Correlation between *DUSP9* and established oncofetal genes in the TCGA‐LIHC cohort. (J) UMAP plot displaying expression levels of *DUSP9* and established oncofetal and stemness‐associated genes in epithelial subclusters in HCC single‐cell RNA‐seq data (GSE16635). The results are presented as mean ± SD and analysed using Student's *t*‐test. Spearman's rank correlation test was used to analyse the correlation between continuous variables. ns*p* > .05; *p < .05; **p < .01; ****p < .0001.

To identify key oncofetal proteins associated with HCC stemness and dedifferentiation, we performed a multi‐omics analysis by integrating data from HCC‐derived and healthy liver‐derived organoids, as well as scRNA‐seq data of hepatoblasts. By intersecting the upregulated genes in HCC‐derived organoids (vs. healthy liver organoids), the dedifferentiation‐associated genes from the CytoTRACE database^18^ and the abovementioned 23 oncofetal genes, we identified five key oncofetal genes: *ASCL4, DUSP9, MCM2, PEG10 and ROBO1* (Figure 3A). We then assessed the expression of these five genes in murine adult liver, fetal liver and Hepa1‐6 cells. Among them, *DUSP9* exhibited the most distinct oncofetal expression pattern, being highly expressed in fetal liver and Hepa1‐6 cells, while showing minimal expression in adult liver (Figure 3B). This oncofetal expression pattern was further confirmed using the GepLiver database,^24^ where *DUSP9* mRNA was significantly elevated in both fetal liver and HCC tissues compared to normal adult liver, in both murine (Figure 3C) and human samples (Figure 3D).

We further investigated the expression dynamics of five oncofetal genes along the differentiation trajectory of murine hepatoblasts using CytoTRACE‐based pseudotime analysis. Specifically, we assessed the correlation between each gene's expression and cell differentiation status. Among the five genes, *Dusp9* exhibited the strongest inverse correlation with cell differentiation, with its expression markedly elevated in poorly differentiated (stem‐like) hepatoblasts (Figure 3E). This relationship was clearly visualized in the pseudotime analysis, where *DUSP9* displayed a distinct negative trend with cell differentiation, indicating that its high expression is a characteristic feature of low‐differentiation (stem‐like) states. In contrast, the other four genes (*Mcm2, Peg10, Robo1*) showed relatively weaker or less consistent correlations with differentiation status (Figure S5A–D). These results highlight *DUSP9* as the most differentiation‐sensitive oncofetal gene among the five candidates, further supporting its potential role in maintaining dedifferentiation and stemness in HCC.

We next systematically evaluated *DUSP9* expression across 12 HCC bulk transcriptomic datasets. Normalized against adjacent non‐tumour tissues (mean expression = 1), *DUSP9* was upregulated in 52.1% of 2048 HCC tumours, whereas only 5.8% of tumours showed downregulation (Figure 3F). Notably, three datasets displayed particularly high frequencies of *DUSP9* upregulation (73.1%, 84.0% and 84.9%). Consistently, *DUSP9* expression was significantly higher in HCC‐derived organoids compared to healthy liver‐derived organoids (Figure 3G). At the protein level, proteomic analysis of 165 paired HCC and adjacent tissues from the CPTAC dataset confirmed that DUSP9 protein was significantly overexpressed in tumour tissues (Figure 3H).

To further establish DUSP9 as an oncofetal protein, we examined its correlation with established oncofetal genes in the TCGA‐LIHC cohort. *DUSP9* displayed significant positive correlations with multiple classical oncofetal genes, including *AFP, GPC3 and IGF2* (Figure 3I), suggesting a strong oncofetal signature. We further explored the scRNA‐seq data of HCC (GSE16635) to elucidate *DUSP9* expression at the single‐cell level. Tumour epithelial cells were further reclustered into two distinct subclusters (Subcluster 1 and Subcluster 2), and *DUSP9* was predominantly expressed in Subcluster 1, which was enriched with other oncofetal and stemness‐related genes such as *AFP, GPC3, IGF2, ANPEP* and *EPCAM* (Figure 3J). Interestingly, *HLF* and *SDC1*, which showed negative correlations with *DUSP9* at the bulk tissue level in the TCGA‐LIHC dataset, were co‐expressed with *DUSP9* in Subcluster 1 at the single‐cell level (Figure S5E). This co‐expression feature at single‐cell level further highlights DUSP9 as a key oncofetal protein associated with stemness features in HCC.

Collectively, these integrated multi‐omic analyses identify DUSP9 as a key oncofetal protein in HCC, characterized by its elevated expression in fetal and tumour tissues, its correlation with dedifferentiation and stemness and its co‐expression with other canonical oncofetal genes.

### 3.5 DUSP9 exhibits an oncofetal expression pattern and correlates with adverse prognosis in hepatocellular carcinoma

**FIGURE 4:**
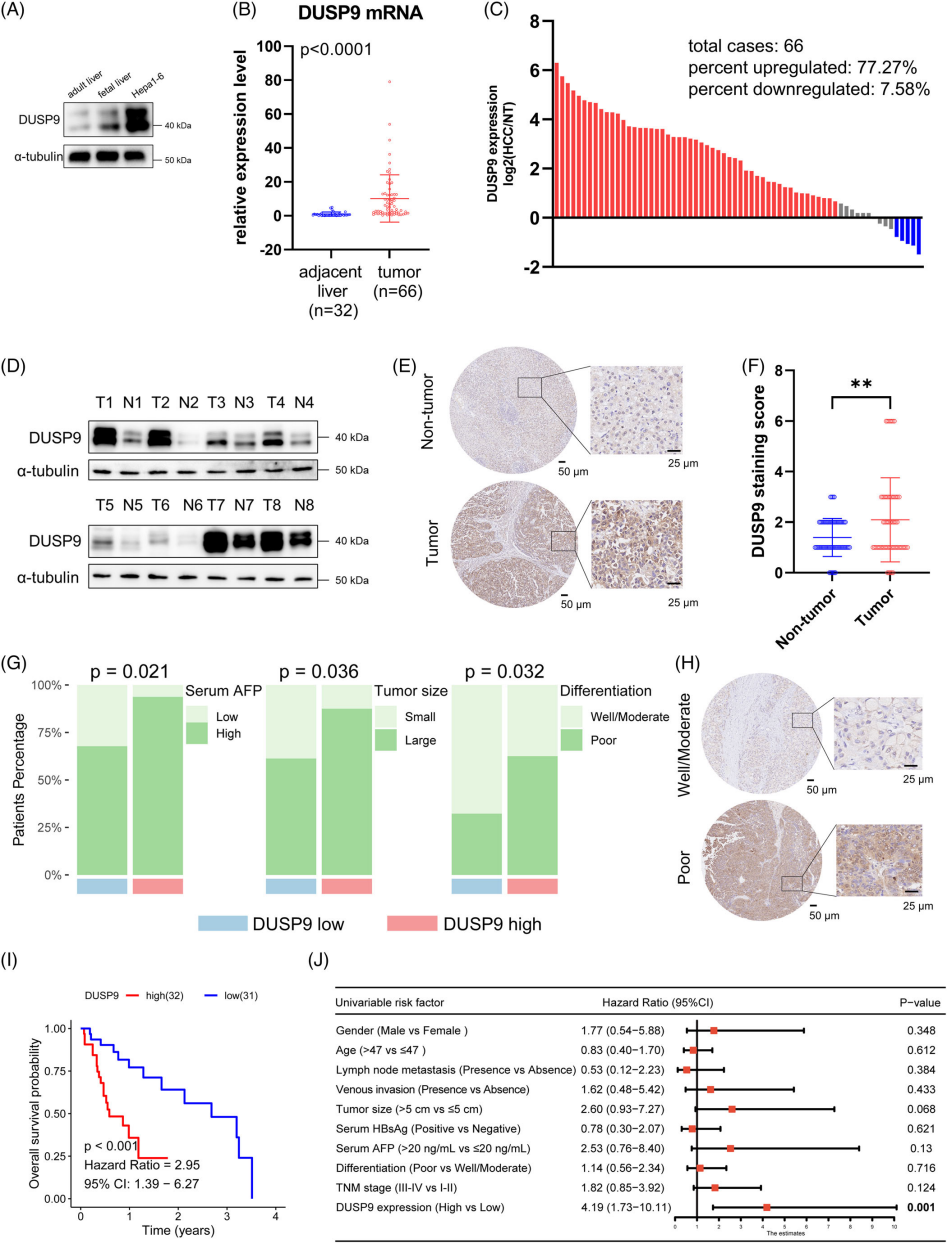
Dual‑specificity phosphatase 9 (DUSP9) exhibits an oncofetal expression pattern and correlates with adverse prognosis in hepatocellular carcinoma. (A) DUSP9 protein expression in murine adult liver, fetal liver and Hepa1‐6 cells. (B) *DUSP9* mRNA expression in HCC tissues (*n* = 66) versus adjacent tissues (*n* = 32). (C) Distribution of *DUSP9* expression changes in HCC tissues, normalized to paired adjacent tissues. (D) Western blot showing DUSP9 protein levels in paired HCC and adjacent tissues. (E) Representative IHC images of DUSP9 expression in HCC and adjacent tissues (*n* = 63; scale bars: 50 and 25 µm). (F) Quantification of DUSP9 IHC staining scores in HCC and paired adjacent tissues. (G) Association between DUSP9 expression and clinical characteristics (serum AFP levels, tumour size, differentiation). (H) Representative IHC staining images of DUSP9 in well/moderately differentiated and poorly differentiated HCC tissues (scale bars: 100 and 20 µm). (I) Kaplan–Meier survival analysis in 63 HCC patients stratified by DUSP9 expression. (J) Forest plot of univariate Cox regression analysis of risk factors for the overall survival in 63 patients. The results are presented as mean ± SD and analysed using Student's *t*‐test. Chi‐square test and Fisher's exact test were used to analyse the correlation between categorical variables. Survival analysis was performed using the log‐rank test. **p < .01. CI, confidence interval.

Having identified DUSP9 as a key oncofetal protein in HCC, we further validated its oncofetal characteristics using animal models and clinical specimens. The murine DUSP9 showed prominent expression in both Hepa1‐6 hepatoma cells and fetal liver tissues compared to healthy adult liver tissues (Figure 4A). In clinical tissue specimens, *DUSP9* expression was significantly higher in HCC tumours compared to adjacent non‐tumour tissues (Figure 4B). Overall, 77.27% of HCC patients exhibited *DUSP9* upregulation, whereas only 7.58% showed downregulation (Figure 4C). Western blot analysis of eight randomly selected paired HCC and adjacent tissues confirmed that DUSP9 was highly expressed in tumour tissues, with low expression in adjacent tissues (Figure 4D). IHC staining of a tissue microarray comprising 63 paired HCC and adjacent tissue samples also demonstrated robust DUSP9 expression in HCC tumours (Figure 4E,F). We next investigated the association between DUSP9 expression and clinicopathological features. High DUSP9 expression was positively correlated with elevated serum AFP levels, large tumour size and poorer differentiation in our HCC tissue microarray cohort (Figure 4G; Table 1). Representative IHC images showed that poorly differentiated tumours exhibited higher DUSP9 expression (Figure 4H). Consistent findings were observed in the TCGA‐LIHC and CHCC cohorts, where high *DUSP9* expression was associated with adverse clinicopathological features, including high serum AFP and poor tumour differentiation (Tables 2 and 3).

**TABLE 1:** Association of dual‑specificity phosphatase 9 (DUSP9) expression with clinicopathologic features in 63 hepatocellular carcinoma (HCC) cases.

Features	Total (*n* = 63)	Low DUSP9 *n* = 31 (49%)	High DUSP9 *n* = 32 (51%)	*p* value
Age, years				.895
≤47 (median)	30 (47.62%)	14 (45.16%)	16 (50.00%)	
>47	33 (52.38%)	17 (54.84%)	16 (50.00%)	
Gender				>.999
Female	9 (14.29%)	4 (12.90%)	5 (15.63%)	
Male	54 (85.71%)	27 (87.10%)	27 (84.38%)	
Serum HBsAg				.237
Negative	8 (12.70%)	6 (19.35%)	2 (6.25%)	
Positive	55 (87.30%)	25 (80.65%)	30 (93.75%)	
Serum AFP (ng/mL)				**.021**
Low (≤20)	12 (19.05%)	10 (32.26%)	2 (6.25%)	
High (>20)	51 (80.95%)	21 (67.74%)	30 (93.75%)	
Differentiation				**.032**
Well/Moderate	33 (52.38%)	21 (67.74%)	12 (37.50%)	
Poor	30 (47.62%)	10 (32.26%)	20 (62.50%)	
Tumour size				**.036**
Small (≤5 cm)	16 (25.40%)	12 (38.71%)	4 (12.50%)	
Large (>5 cm)	47 (74.60%)	19 (61.29%)	28 (87.50%)	
TNM stage (AJCC)				.341
I–II	40 (63.49%)	22 (70.97%)	18 (56.25%)	
III–IV	23 (36.51%)	9 (29.03%)	14 (43.75%)	
Lymph node metastasis				.670
Absence	55 (87.30%)	26 (83.87%)	29 (90.63%)	
Presence	8 (12.70%)	5 (16.13%)	3 (9.38%)	
Venous invasion				.371
Absence	58 (92.06%)	30 (96.77%)	28 (87.50%)	
Presence	5 (7.94%)	1 (3.23%)	4 (12.50%)	

*Note*: Statistical significance (*p* < .05) is shown in bold. Chi‐square test and Fisher's exact test.

**TABLE 2:** Association of dual‑specificity phosphatase 9 (*DUSP9*) expression with clinicopathologic features in the Cancer Genome Atlas (TCGA) dataset.

Features	Total (*n* = 63)	Low *DUSP9* *n* = 172 (90.1%)	High *DUSP9* *n* = 19 (9.9%)	*p* value
Age, years				.577
≤61 (median)	107 (56.0%)	98 (57%)	9 (47.4%)	
>61	84 (44.0%)	74 (43%)	10 (52.6%)	
Gender				.151
Female	58 (30.4%)	49 (28.5%)	9 (47.4%)	
Male	133 (69.6%)	123 (71.5%)	10 (52.6%)	
Grade				**.024**
G1	15 (7.9%)	15 (8.7%)	0 (0%)	
G2	90 (47.1%)	85 (49.4%)	5 (26.3%)	
G3/G4	86 (45.0%)	72 (41.9%)	14 (73.7%)	
Serum AFP (ng/mL)				**8.4e–6**
Low (≤20)	107 (56.0%)	106 (61.6%)	1 (5.3%)	
High (>20)	84 (44.0%)	66 (38.4%)	18 (94.7%)	
TNM stage (AJCC)				.353
I–II	160 (83.8%)	146 (84.9%)	14 (73.7%)	
III–IV	31 (16.2%)	26 (15.1%)	5 (26.3%)	
Vascular invasion				.169
Absence	132 (69.1%)	122 (70.9%)	10 (52.6%)	
Presence	59 (30.9%)	50 (29.1%)	9 (47.4%)	
Child–Pugh stage				>.999
A	174 (91.1%)	157 (91.3%)	17 (89.5%)	
B/C	17 (8.9%)	15 (8.7%)	2 (10.5%)	

*Note*: Statistical significance (*p* < .05) is shown in bold. Chi‐square test and Fisher's exact test.

**TABLE 3:** Association of dual‑specificity phosphatase 9 (*DUSP9*) expression with clinicopathologic features in CHCC dataset.

Features	Total (*n* = 63)	Low *DUSP9* *n* = 77 (48.4%)	High *DUSP9* *n* = 82 (51.6%)	*p* value
Age, years				.184
≤54 (median)	84 (52.8%)	36 (46.8%)	48 (58.5%)	
>54	75 (47.2%)	41 (53.2%)	34 (41.5%)	
Gender				.159
Female	31 (19.5%)	11 (14.3%)	20 (24.4%)	
Male	128 (80.5%)	66 (85.7%)	62 (75.6%)	
Cirrhosis				.432
Absence	47 (29.6%)	20 (26%)	27 (32.9%)	
Presence	112 (70.4%)	57 (74%)	55 (67.1%)	
Tumour number				.134
Single	117 (73.6%)	52 (67.5%)	65 (79.3%)	
Multiple	42 (26.4%)	25 (32.5%)	17 (20.7%)	
Serum AFP (ng/mL)				**.0006**
Low (≤20)	58 (36.5%)	39 (50.6%)	19 (23.2%)	
High (>20)	101 (63.5%)	38 (49.4%)	63 (76.8%)	
Tumour encapsulation				.546
Absence	48 (30.2%)	21 (27.3%)	27 (32.9%)	
Presence	111 (69.8%)	56 (72.7%)	55 (67.1%)	
Tumour size				.136
Small (≤5 cm)	76 (47.8%)	42 (54.5%)	34 (41.5%)	
Large (>5 cm)	83 (52.2%)	35 (45.5%)	48 (58.5%)	
TNM stage (AJCC)				.374
I–II	105 (66.0%)	54 (70.1%)	51 (62.2%)	
III–IV	54 (34.0%)	23 (29.9%)	31 (37.8%)	
Lymph node metastasis				>.999
Absence	157 (98.7%)	76 (98.7%)	81 (98.8%)	
Presence	2 (1.3%)	1 (1.3%)	1 (1.2%)	
Tumour purity				.962
Low/Middle (≤.9)	119 (74.8%)	57 (74%)	62 (75.6%)	
High (>.9)	40 (25.2%)	20 (26%)	20 (24.4%)	
Tumour thrombus				.097
Absence	122 (76.7%)	64 (83.1%)	58 (70.7%)	
Presence	37 (23.3%)	13 (16.9%)	24 (29.3%)	
BCLC stage				.074
A	68 (42.8%)	39 (50.6%)	29 (35.4%)	
B/C	91 (57.2%)	38 (49.4%)	53 (64.6%)	

*Note*: Statistical significance (*p* < .05) is shown in bold. Chi‐square test and Fisher's exact test.

K–M survival analysis revealed that high DUSP9 expression predicted significantly shorter OS (HR = 2.95, 95% CI = 1.39–6.27) (Figure 4I), and this result was further supported by univariate Cox regression analysis (Figure 4J). Public datasets yielded similar conclusions, with consistent findings in univariate and multivariate Cox regression analyses in three datasets (Figure S6A–C), as well as Kaplan–Meier survival analysis in four datasets (Figure S6D).

Collectively, these findings demonstrate that DUSP9 exhibits an oncofetal expression pattern and is associated with adverse prognosis in patients with HCC.

### 3.6 DUSP9 promotes stemness of HCC cells in vitro and in vivo

Given the observed oncofetal characteristics of DUSP9 and its positive correlation with stemness‐related genes in HCC, we performed systematic in vitro and in vivo experiments to investigate its functional role in regulating stemness in HCC cells.

**FIGURE 5:**
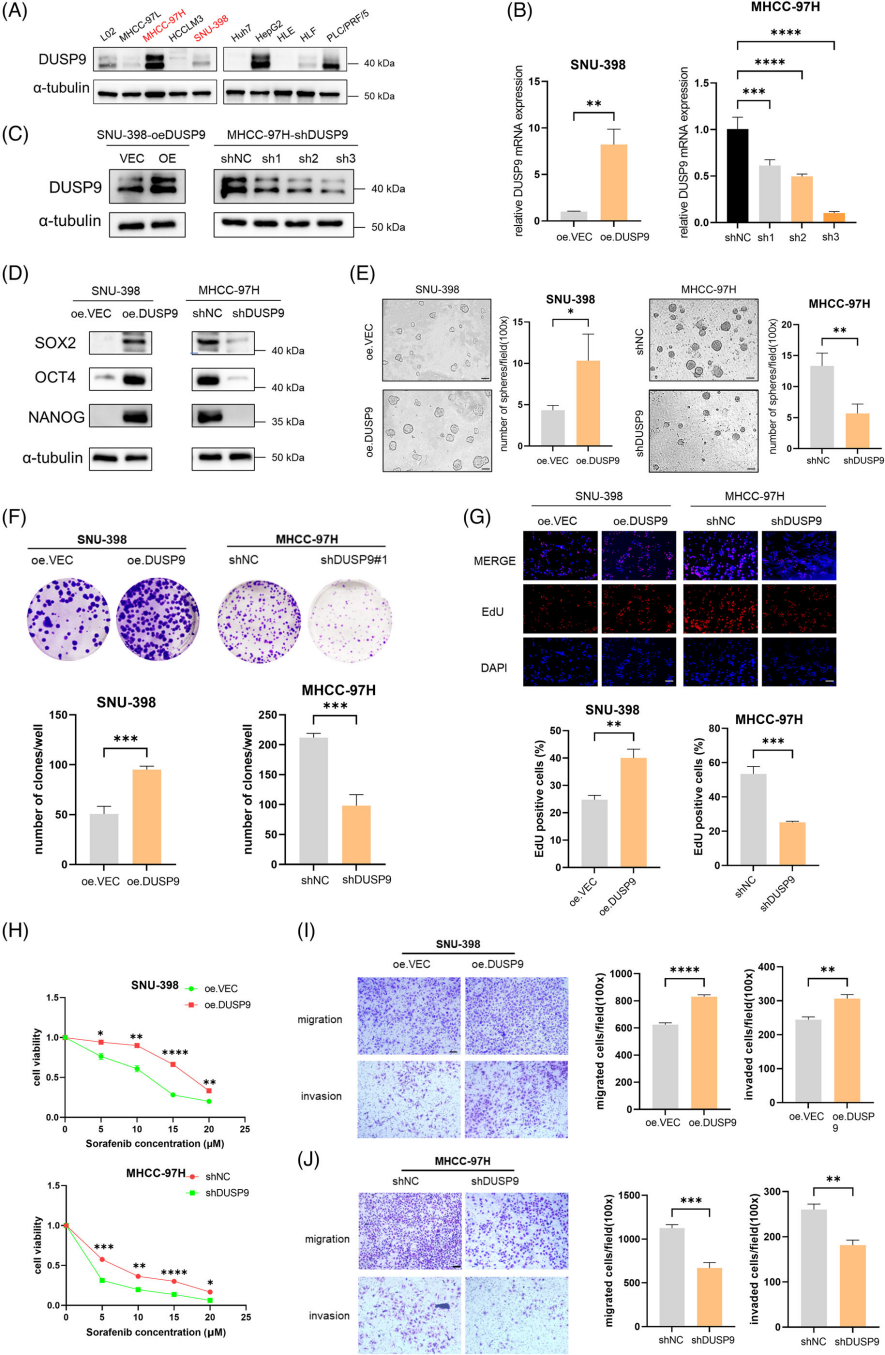
Dual‑specificity phosphatase 9 (DUSP9) promotes stemness of HCC cells in vitro. (A) DUSP9 protein expression in L02 and hepatocellular carcinoma (HCC) cell lines. (B and C) Validation of DUSP9 overexpression and knockdown by quantitative real‐time polymerase chain reaction (qRT‐PCR) (B) and Western blot (C). (D) Western blot showing SOX2, OCT4 and NANOG expression in DUSP9‐modulated HCC cells. (E) Sphere formation assay assessing self‐renewal ability (scale bar: 100 µm). (F) Colony formation assay of DUSP9‐overexpressing and knockdown cells. (G) EdU proliferation assay of DUSP9‐modulated HCC cells. (H) Sorafenib resistance assay showing sensitivity of DUSP9‐modulated cells to sorafenib. (I and J) Transwell migration and invasion assays of DUSP9‐overexpressing SNU‐398 (I) and DUSP9‐knockdown MHCC‐97H cells (J). The results are presented as mean ± SD and analysed using Student's *t*‐test. *p < .05; **p < .01; ***p < .001; ****p < .0001.

Western blot analysis identified MHCC‐97H (high DUSP9 expression) and SNU‐398 (low DUSP9 expression) as representative cell lines for subsequent functional studies (Figure 5A). Using lentiviral transduction, we established stable cell lines: SNU‐398‐oe.VEC (empty vector control), SNU‐398‐oe.DUSP9 (DUSP9 overexpression), MHCC‐97H‐shNC (non‐targeting control) and MHCC‐97H‐shDUSP9‐1/2/3 (DUSP9 knockdown). Transfection efficiency was confirmed by qRT‐PCR (Figure 5B) and Western blot (Figure 5C). Among the knockdown constructs, MHCC‐97H‐shDUSP9‐3 exhibited the highest knockdown efficiency and was selected for subsequent functional assays.

We observed that manipulation of DUSP9 expression led to corresponding changes in the expression of core cancer stemness genes, including SOX2, NANOG and OCT4 (Figure 5D). Sphere formation assay revealed that DUSP9 overexpression significantly enhanced the self‐renewal capacity of HCC cells, whereas knockdown of DUSP9 impaired this ability (Figure 5E). Furthermore, colony formation, EdU proliferation and CCK8 proliferation assays consistently demonstrated that DUSP9 promotes HCC cell proliferation, whereas its knockdown suppresses it (Figure 5F,G; Figure S7A). To assess drug resistance, we treated HCC cells with various concentrations of sorafenib for 24 h. DUSP9‐overexpressing SNU‐398 cells and MHCC‐97H‐shNC controls displayed greater resistance to sorafenib compared to their respective comparative group (SNU‐398‐oe.VEC and MHCC‐97H‐shDUSP9) (Figure 5H). Long‐term colony formation assays under continuous sorafenib treatment demonstrated that DUSP9‐overexpressing SNU‐398 cells showed enhanced colony survival compared to vector controls, whereas DUSP9‐knockdown MHCC‐97H cells exhibited reduced colony survival (Figure S7B). Transwell migration and invasion assays further revealed that DUSP9 expression enhances the metastatic potential of HCC cells (Figure 5I,J).

**FIGURE 6:**
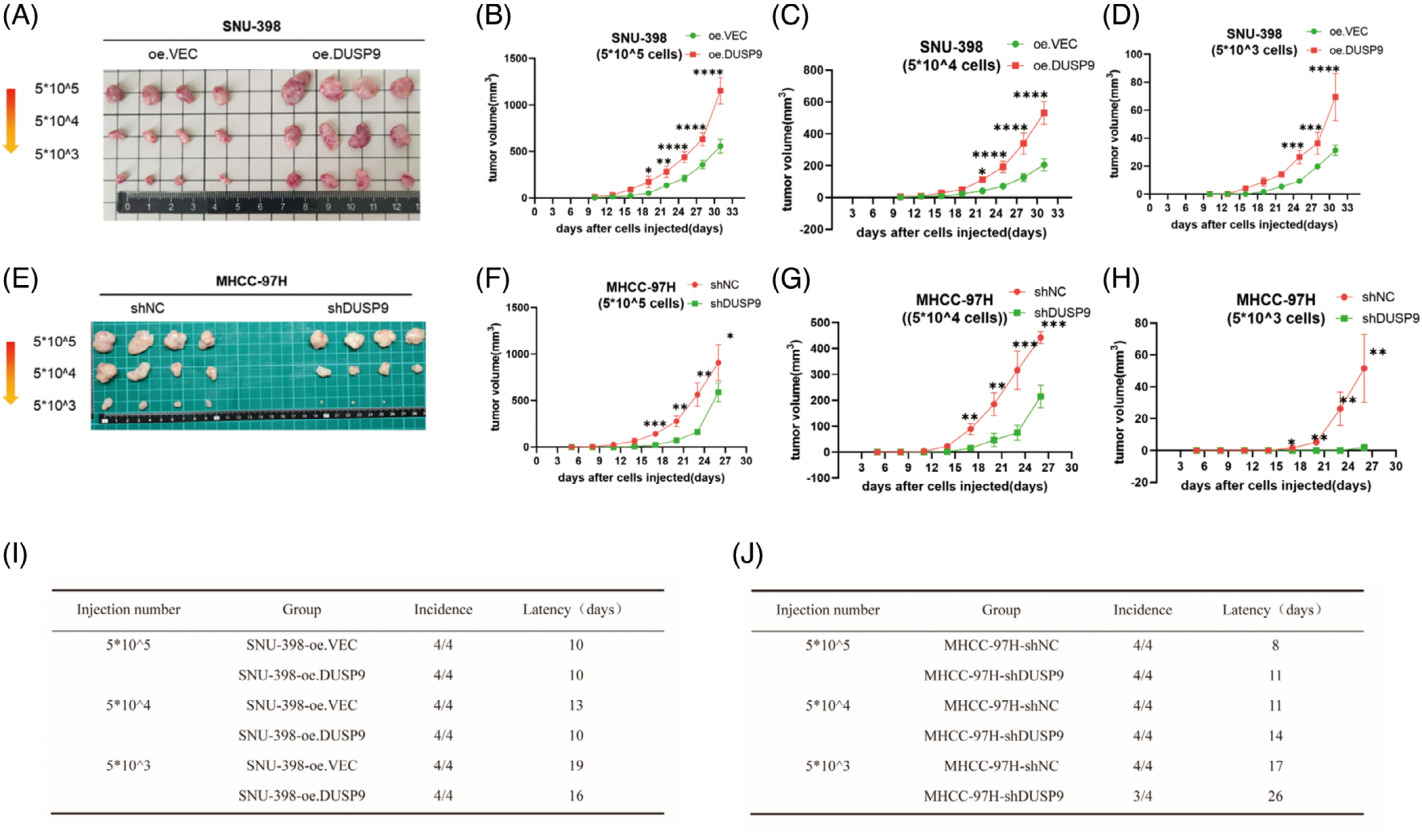
Dual‑specificity phosphatase 9 (DUSP9) promotes stemness of HCC cells in vivo. (A) Gross images of subcutaneous tumours derived from SNU‐398‐oe.VEC and SNU‐398‐oe.DUSP9 cells implanted in BALB/c nude mice (*n* = 4) at varying cell concentrations. (B–D) Tumour growth curves of DUSP9‐overexpressing versus control cells at inoculation doses of 5 × 10^5^ (B), 5 × 10^4^ (C) and 5 × 10^3^ (D) cells. (E) Gross images of subcutaneous tumours derived from MHCC‐97H‐shNC and MHCC‐97H‐shDUSP9 cells implanted in BALB/c nude mice (*n* = 4) at varying cell concentrations. (F–H) Tumour growth curves of DUSP9‐knockdown versus control cells at inoculation doses of 5 × 10^5^ (F), 5 × 10^4^ (G) and 5 × 10^3^ (H) cells. (I and J) Tumour incidence and tumour latency in mice injected with DUSP9‐overexpressing SNU‐398 (I) and DUSP9‐knockdown MHCC‐97H (J) cells at different inoculation concentrations. The results are presented as mean ± SD and analysed using Student's *t*‐test. *p < .05; **p < .01; ***p < .001; ****p < .0001.

To further evaluate the effects of DUSP9 on HCC stemness in vivo, we conducted limiting dilution assays in BALB/c nude mice. SNU‐398 cells with DUSP9 overexpression (SNU‐398‐oe.DUSP9) exhibited enhanced tumour growth compared to vector control cells (SNU‐398‐oe.VEC), as evidenced by faster tumour onset and increased tumour volume, though both groups formed tumours across all inoculation doses (5 × 10^5^, 5 × 10^4^ and 5 × 10^3^ cells; Figure 6A–D,I). This suggests that lower cell numbers would be needed to demonstrate tumour‐initiating frequency differences in SNU‐398 cells, likely due to their inherently high tumorigenic capacity. However, DUSP9 knockdown in MHCC‐97H cells (MHCC‐97H‐shDUSP9) reduced tumourigenicity, with delayed tumour formation, smaller tumour size and lower tumour incidence compared to the control group (MHCC‐97H‐shNC; Figure 6E–H,J). To provide more direct evidence of stemness effects, we performed ALDH activity assays. The results demonstrated that DUSP9 overexpression in SNU‐398 cells significantly increased ALDH activity, whereas DUSP9 knockdown in MHCC‐97H cells reduced ALDH activity. These findings demonstrate that DUSP9 promotes both tumour growth capacity and stem‐like properties of HCC cells (Figure S7C).

Taken together, these findings demonstrated that DUSP9 promotes stemness‐related properties in HCC cells, including self‐renewal, proliferation, sorafenib resistance, migration and invasion.

### 3.7 DUSP9 facilitates lipid metabolism via SCD upregulation

**FIGURE 7:**
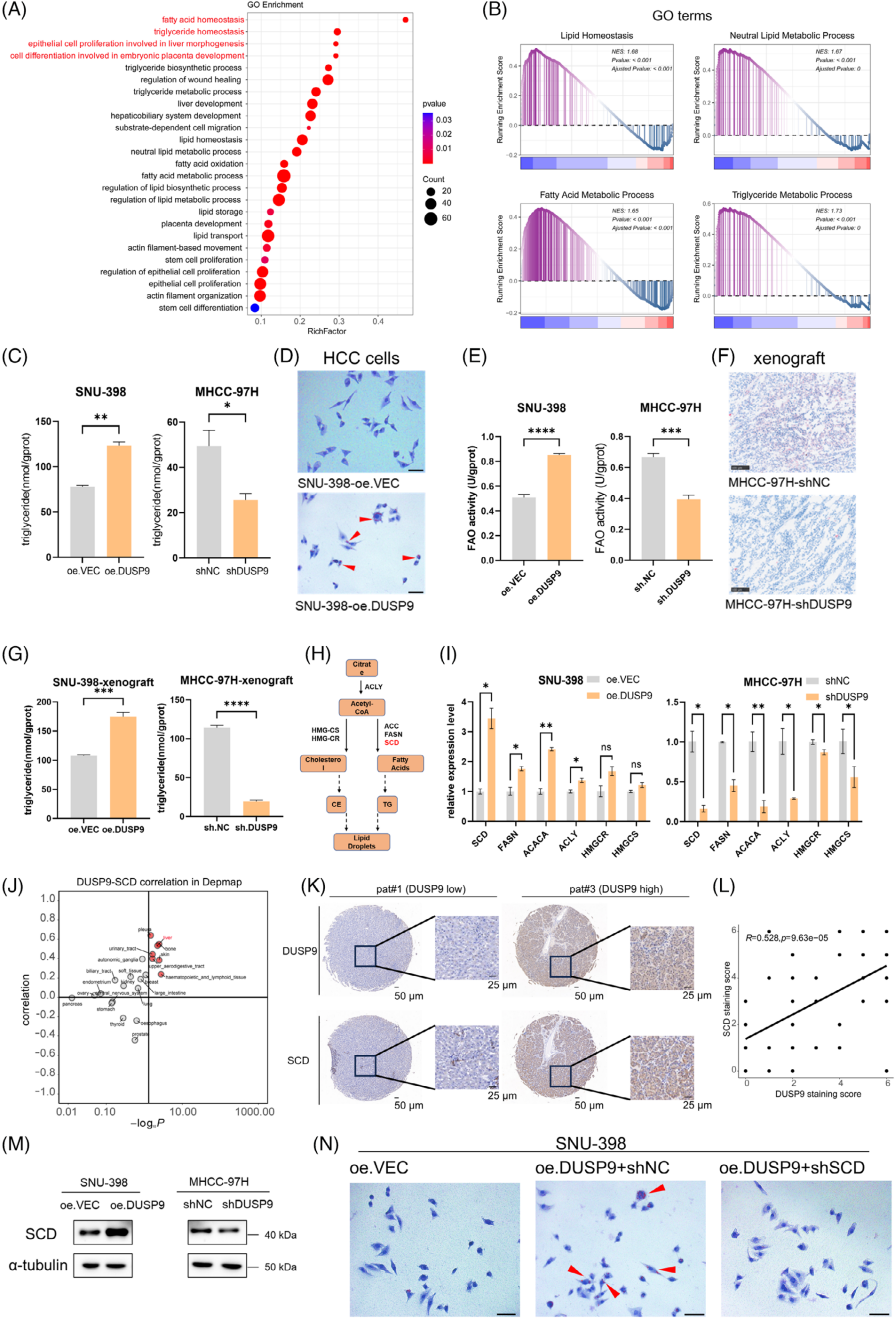
Dual‑specificity phosphatase 9 (DUSP9) facilitates lipid metabolism via stearoyl‑CoA desaturase (SCD) upregulation. (A) Gene Ontology (GO) enrichment of *DUSP9*‐positive versus *DUSP9*‐negative hepatocellular carcinoma (HCC) cells in GSE166635. (B) GSEA analysis showing activation of lipid metabolism‐related pathways in *DUSP9*‐positive HCC cells. (C) Triglyceride quantification in DUSP9‐modulated cells. (D) Oil Red O staining of DUSP9‐overexpressing SNU‐398 cells (scale bar: 20 µm). (E) Fatty acid oxidation (FAO) activities in DUSP9‐modulated cells. (F) Oil Red O staining in subcutaneous xenografts from DUSP9‐knockdown MHCC‐97H cells (scale bar: 100 µm). (G) Triglyceride quantification in subcutaneous xenografts from DUSP9‐modulated cells. (H) Diagram of key enzymes in lipid metabolism. (I) Quantitative real‐time polymerase chain reaction (qRT‐PCR) of lipid metabolism genes in DUSP9‐modulated cells. (J) Correlation between *DUSP9* and *SCD* expression in DepMap pan‐cancer transcriptomic data. (K) Representative IHC images of DUSP9 and SCD expression in HCC tumours (scale bars: 50 and 25 µm). (L) Correlation between DUSP9 and SCD staining scores in HCC microarray (*n* = 49). (M) Western blot of SCD protein levels in DUSP9‐modulated cells. (N) Oil Red O staining in DUSP9–SCD sequential‐modulated SNU‐398 cells (scale bar: 20 µm). The results are presented as mean ± SD and analysed using Student's *t*‐test. Spearman's rank correlation test was used to analyse the correlation between continuous variables. ns*p* > .05; *p < .05; **p < .01.

To elucidate the mechanism by which DUSP9 promotes stemness in HCC, we conducted differential gene expression and functional enrichment analyses by comparing *DUSP9*‐positive (non‐zero expression) and *DUSP9*‐negative (zero expression) HCC cells. GO enrichment analysis revealed significant upregulation of genes involved in lipid metabolism and cell differentiation pathways in *DUSP9*‐positive cells (Figure 7A). In line with these findings, GSEA analysis also identified lipid homeostasis as a significantly enriched pathway in *DUSP9*‐positive HCC cells (Figure 7B).

To investigate whether *DUSP9* expression correlates with specific metabolic changes, we performed metabolite correlation analysis across HCC cell lines. The results demonstrated a strong positive association between *DUSP9* expression and the abundance of various triglyceride species (Figure S8A). Moreover, scFEA revealed elevated fatty acid biosynthesis activity in the *DUSP9*‐high tumour subcluster (Subcluster 1) (Figure S8B).^19^ Moreover, triglyceride levels in HCC cells were significantly increased or decreased following DUSP9 overexpression or knockdown, respectively (Figure 7C). Oil Red O staining confirmed enhanced lipid droplet accumulation in DUSP9‐overexpressing SNU‐398 cells (Figure 7D). We specifically investigated the functional aspect of lipid metabolism by assessing the FAO capacity in cells with altered DUSP9 expression, as our enrichment analysis identified fatty acid‐related pathways as significantly activated in *DUSP9*‐positive cells. The FAO assay results demonstrated that DUSP9 overexpression in SNU‐398 cells significantly enhanced their FAO ability, whereas DUSP9 knockdown in MHCC‐97H cells resulted in a reduction in FAO activity (Figure 7E). DUSP9‐knockdown MHCC‐97H‐derived xenografts exhibited reduced lipid deposition (Figure 7F). Triglyceride quantification assays were also performed on xenograft tumour tissues. The results demonstrated that DUSP9‐overexpressing SNU‐398 xenografts exhibited elevated triglyceride levels compared to vector control tumours. Conversely, DUSP9‐knockdown MHCC‐97H xenografts showed reduced triglyceride content compared to control tumours (Figure 7G). This functional validation evidence supported that DUSP9 actively regulates FAO, which provides energy and biosynthetic precursors essential for CSC maintenance and proliferation.

To pinpoint the enzymes mediating DUSP9‐driven lipid remodelling, we performed qRT‐PCR to measure the expression of key enzymes in fatty acid metabolism (*ACLY, ACACA, FASN, SCD*) and cholesterol synthesis (*HMGCR, HMGCS*) (Figure 7H). Among these, *SCD* exhibited the most robust response to DUSP9 modulation, suggesting it may be a critical downstream effector (Figure 7I). We next evaluated the *DUSP9–SCD* expression correlation across large‐scale datasets. In the TCGA‐LIHC dataset, a modest positive correlation was observed (*r* = .173) (Figure S8C). However, in pan‐cancer cell line datasets, this correlation was much stronger (*r* = .572) (Figure 7J; Figure S8D). The LIMORE dataset, which includes transcriptomic data from 81 HCC cell lines,^25^ further validated this finding with a robust correlation (*r* = .58) (Figure S8E). This transcriptomic discrepancy between tissues and cell lines may reflect cell‐type‐specific expression patterns of *DUSP9*. We then performed IHC co‐staining for DUSP9 and SCD on the same tissue microarray. Representative images showed the visual evidence of DUSP9 and SCD expression in the same HCC tissue section (Figure 7K). Quantitative analysis demonstrated significant positive correlation between DUSP9 and SCD protein expression in HCC tumours (Figure 7L).

SCD is a key enzyme involved in lipid metabolism, catalysing the biosynthesis of monounsaturated fatty acids (MUFAs), which are essential components of membrane phospholipids, cholesterol esters, triglycerides and signalling lipids. Thus, SCD plays a central role in lipid homeostasis and has emerged as a promising therapeutic target in cancer.^14^ Moreover, SCD has been reported to regulate CSC functions in colorectal, gastric and oral squamous carcinomas.^15^, ^16^, ^17^ *SCD* upregulation was observed in 54.4% of 1968 HCC tumours, and high *SCD* expression was associated with shorter OS (Figure S8F,G). Western blot further confirmed that DUSP9 positively regulates SCD protein expression (Figure 7M). Importantly, SCD knockdown in SNU‐398‐oe.DUSP9 cells markedly reduced DUSP9‐induced lipid droplet accumulation (Figure 7N; Figure S8H). We performed FAO activity assays in cells with sequential modulation of DUSP9 and SCD. SCD knockdown attenuated DUSP9‐induced FAO enhancement in SNU‐398 cells, whereas SCD overexpression restored FAO capacity in DUSP9‐knockdown MHCC‐97H cells (Figure S8I). These results confirm SCD as a critical downstream effector of DUSP9 in regulating lipid metabolism.

Taken together, these findings demonstrate that DUSP9 facilitates lipid metabolism in HCC by upregulating SCD.

### 3.8 DUSP9 drives HCC stemness by upregulating SCD

**FIGURE 8:**
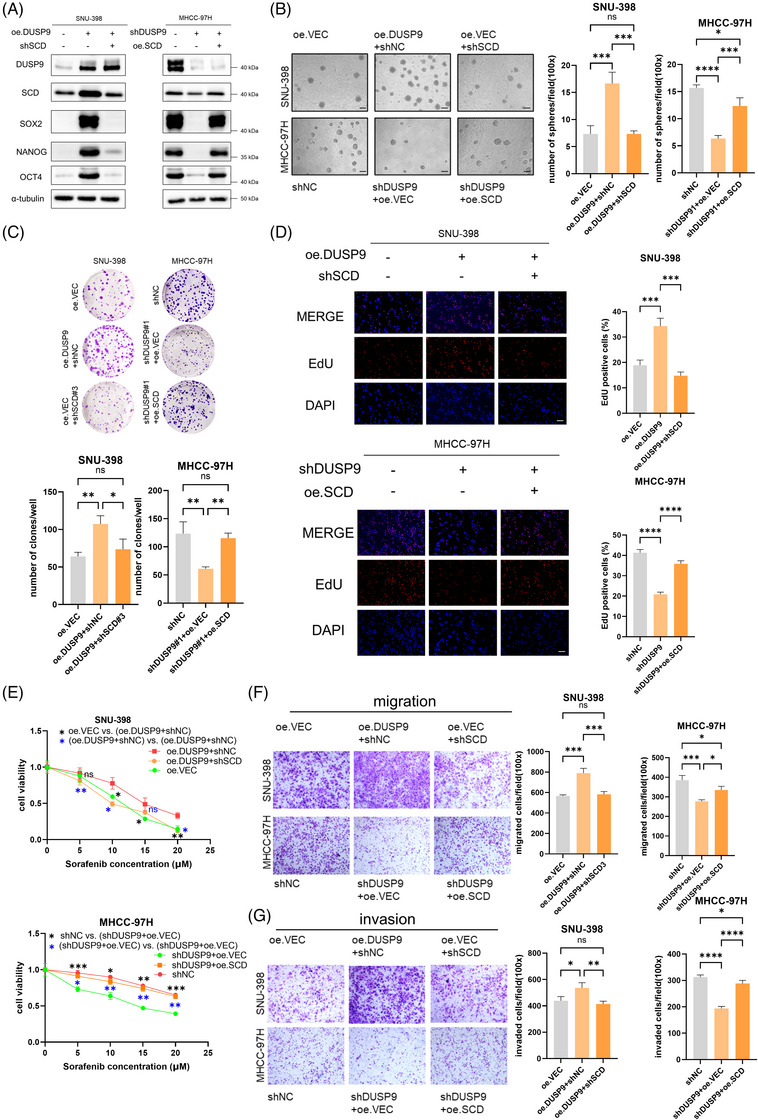
Dual‑specificity phosphatase 9 (DUSP9) drives HCC stemness by upregulating stearoyl‑CoA desaturase (SCD). (A) Western blot of stemness genes (SOX2, OCT4, NANOG) in DUSP9–SCD sequential‐modulated hepatocellular carcinoma (HCC) cells. (B) Sphere formation assay of DUSP9–SCD sequential‐modulated HCC cells (scale bar: 100 µm). (C–E) Colony formation (C), EdU proliferation (D) and sorafenib resistance (E), Transwell migration (F) and invasion (G) assays in DUSP9–SCD sequential‐modulated HCC cells. The results are presented as mean ± SD and analysed using Student's *t*‐test or one‐way ANOVA followed by Tukey's post hoc test for multiple comparisons as appropriate. Spearman's rank correlation test was used to analyse the correlation between continuous variables. ns*p* > .05; *p < .05; **p < .01; ***p < .001; ****p < .0001.

Recent advances in proteomics and metabolomics have underscored the pivotal roles of lipid metabolism in regulating CSC fate.^26^ Given our previous findings that DUSP9 reprogrammes lipid metabolism via upregulating SCD, we next investigated whether SCD‐mediated lipid remodelling is essential for DUSP9‐driven stemness in HCC. To address this, we performed sequential modulation of SCD in the context of DUSP9 overexpression or knockdown. Specifically, SCD was silenced in DUSP9‐overexpressing SNU‐398 cells and overexpressed in DUSP9‐knockdown MHCC‐97H cells via lentiviral transduction. Western blot analysis confirmed the effective modulation of SCD expression and further demonstrated that the DUSP9–SCD axis directly influences the expression of canonical stemness genes (Figure 8A).

To evaluate the functional consequences, we conducted a series of in vitro assays. In the sphere formation assay, downregulation of SCD significantly attenuated the enhanced self‐renewal capacity conferred by DUSP9 overexpression. Conversely, SCD upregulation rescued the impaired self‐renewal in DUSP9‐knockdown cells (Figure 8B). Consistently, SCD silencing in SNU‐398‐oe.DUSP9 cells markedly diminished DUSP9‐induced colony formation, proliferation, sorafenib resistance, as well as migration and invasion capabilities (Figure 8C–G; Figure S9A,B). In contrast, SCD restoration in MHCC‐97H‐shDUSP9 cells rescued these suppressed phenotypes, reinstating colony formation, growth, drug resistance and metastatic potential (Figure 8C–G; Figure S9A,B).

Together, these results strongly indicate that SCD‐mediated lipid metabolism is a critical downstream effector through which DUSP9 enhances HCC stemness. This highlights a functional DUSP9–SCD–lipid axis that governs both metabolic and malignant reprogramming in HCC.

### 3.9 DUSP9 upregulates SCD through the ERK1/2‐PPARG axis to promote HCC stemness

We further explored the upstream regulatory mechanism of SCD activation by DUSP9. Prior studies have shown that DUSP9 negatively regulates the MAPK pathway by dephosphorylating ERK1/2.^27^ Building on our observation that DUSP9 transcriptionally enhances the expression of a variety of lipid metabolism enzymes, we hypothesized that DUSP9 promotes lipid metabolism by modulating key transcription factors of lipid metabolism downstream of ERK1/2.

**FIGURE 9:**
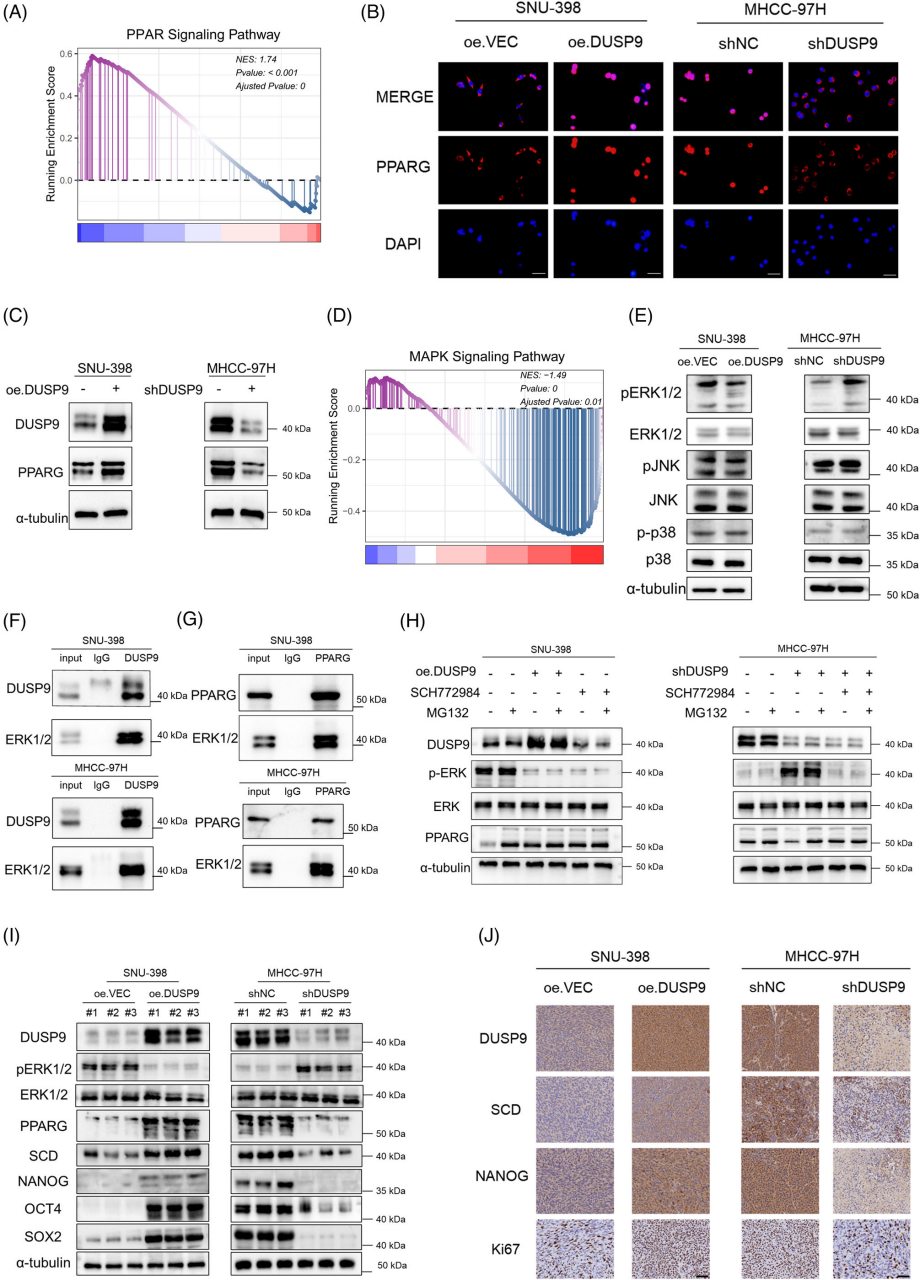
Dual‑specificity phosphatase 9 (DUSP9) upregulates stearoyl‑CoA desaturase (SCD) through the ERK1/2‐PPARG axis to promote hepatocellular carcinoma (HCC) stemness. (A) GSEA analysis of the peroxisome proliferator‐activated receptor (PPAR) signalling pathway in *DUSP9*‐positive HCC cells. (B) Immunofluorescence showing PPARG subcellular localization in DUSP9‐modulated HCC cells (scale bar: 20 µm). (C) Western blot of PPARG expression in DUSP9‐modulated HCC cells. (D) GSEA analysis of the MAPK signalling pathway in *DUSP9*‐positive HCC cells. (E) Western blot of MAPK pathway components (ERK1/2, p38, JNK1/2/3) and their phosphorylation levels in DUSP9‐modulated cells. (F) Co‐immunoprecipitation assays showing interactions of DUSP9 with ERK1/2. (G) Co‐immunoprecipitation assays showing interactions of PPARG with ERK1/2. (H) Effects of ERK1/2 inhibitor SCH772984 and proteasome inhibitor MG132 on PPARG expression in DUSP9‐modulated HCC cells. (I) Western blot showing DUSP9, pERK1/2, ERK1/2, PPARG, SCD, NANOG, OCT4 and SOX2 expression in subcutaneous xenografts from DUSP9‐modulated HCC cells. (J) IHC images showing DUSP9, SCD, NANOG and Ki67 expression in subcutaneous xenografts from DUSP9‐modulated HCC cells (scale bars: 100 µm).

GSEA enrichment analysis of single‐cell transcriptomic data revealed a significant activation of the peroxisome proliferator‐activated receptor (PPAR) signalling pathway in *DUSP9*‐positive HCC cells (Figure 9A). Among the PPAR isoforms, PPARG—a nuclear receptor pivotal in hepatic lipid metabolism^28^—was prioritized for our further investigation. First, the BioGRID database (https://thebiogrid.org/) indicated that ERK2 directly interacts with PPARG, but not with other PPAR isoforms. Second, TCGA correlation analysis demonstrated that *PPARG* exhibits stronger correlation with *SCD* expression in HCC compared to other PPAR isoforms (Figure S10A). Moreover, PPARG is a known transcriptional activator of SCD and other lipid enzymes.^29^ Supporting this, public datasets show that inhibition or knockout of PPARG in bladder carcinoma cells significantly reduces *SCD* expression (Figure S10B). To determine whether DUSP9 regulates PPARG in HCC, we assessed PPARG expression at both mRNA and protein levels. qRT‐PCR analysis revealed no significant changes in *PPARG* mRNA upon DUSP9 modulation (Figure S10C). However, DUSP9 overexpression markedly increased PPARG protein levels and promoted its nuclear localization, suggesting a post‐transcriptional regulation mechanism (Figure 9B,C). ChIP‐qPCR assays confirmed that PPARG directly binds to the *SCD* promoter region in HCC cells (Figure S10D). PPARG knockdown significantly reduced SCD expression and phenocopied the effects of SCD inhibition on stem‐like properties in MHCC‐97H cells. Specifically, PPARG knockdown reduced sphere formation capacity, proliferation, sorafenib resistance, migration and invasion abilities (Figure S11A–G).

Literature mining indicated that ERK1/2‐mediated phosphorylation promoted PPARG ubiquitination and degradation,^30^, ^31^ implying that DUSP9 may stabilize PPARG via ERK1/2 inhibition. Supporting this, GSEA analysis demonstrated significant inactivation of MAPK signalling in *DUSP9*‐positive HCC cells (Figure 9D), and *DUSP9* expression negatively correlated with ERK1/2 phosphorylation across HCC cell lines in the DepMap dataset (Figure S12A). Western blot assays further confirmed that DUSP9 overexpression reduces phosphorylated ERK1/2 levels, whereas its knockdown increases ERK1/2 phosphorylation (Figure 9E). Furthermore, treatment with ERK1/2 inhibitor SCH772984 independently upregulated SCD protein expression in SNU‐398 cells (Figure S12B).

Co‐immunoprecipitation (Co‐IP) assays and molecular docking validated the physical interaction between DUSP9 and ERK1/2, as well as between ERK1/2 and PPARG (Figure 9F,G; Figure S12C,D), reinforcing the proposed mechanistic link. Moreover, pharmacological inhibition of ERK1/2 (using SCH772984) or proteasome inhibition (MG132) restored PPARG protein levels in DUSP9‐modulated HCC cells. Specifically, ERK1/2 or proteasome inhibition significantly rescued the DUSP9‐knockdown‐induced impaired PPARG protein expression in MHCC‐97H cells, confirming the involvement of this post‐translational regulatory axis (Figure 9H).

To further elucidate the molecular mechanisms underlying DUSP9‐induced stemness, we performed Western blot on xenograft tumours derived from DUSP9‐modulated HCC cells. The results confirmed that DUSP9 overexpression led to the deactivation of the ERK1/2 signalling pathway, as indicated by reduced p‐ERK1/2 levels, and enhanced expression of PPARG and SCD, consistent with our proposed DUSP9–ERK1/2–PPARG–SCD axis (Figure 9I). In addition, key stemness markers, including NANOG, OCT4 and SOX2, were markedly upregulated in DUSP9‐overexpressing tumours, whereas their expression was significantly reduced in DUSP9‐knockdown tumours, further supporting the role of DUSP9 in promoting stemness. Moreover, immunohistochemical staining of xenograft tumours further validated these findings. DUSP9‐overexpressing tumours showed higher levels of DUSP9, SCD, NANOG and Ki67, whereas DUSP9‐knockdown tumours displayed reduced expression of these markers (Figure 9J). These results are consistent with our WB analysis and provide additional evidence that DUSP9 enhances the stemness and proliferative capacity of HCC cells in vivo.

**FIGURE 10:**
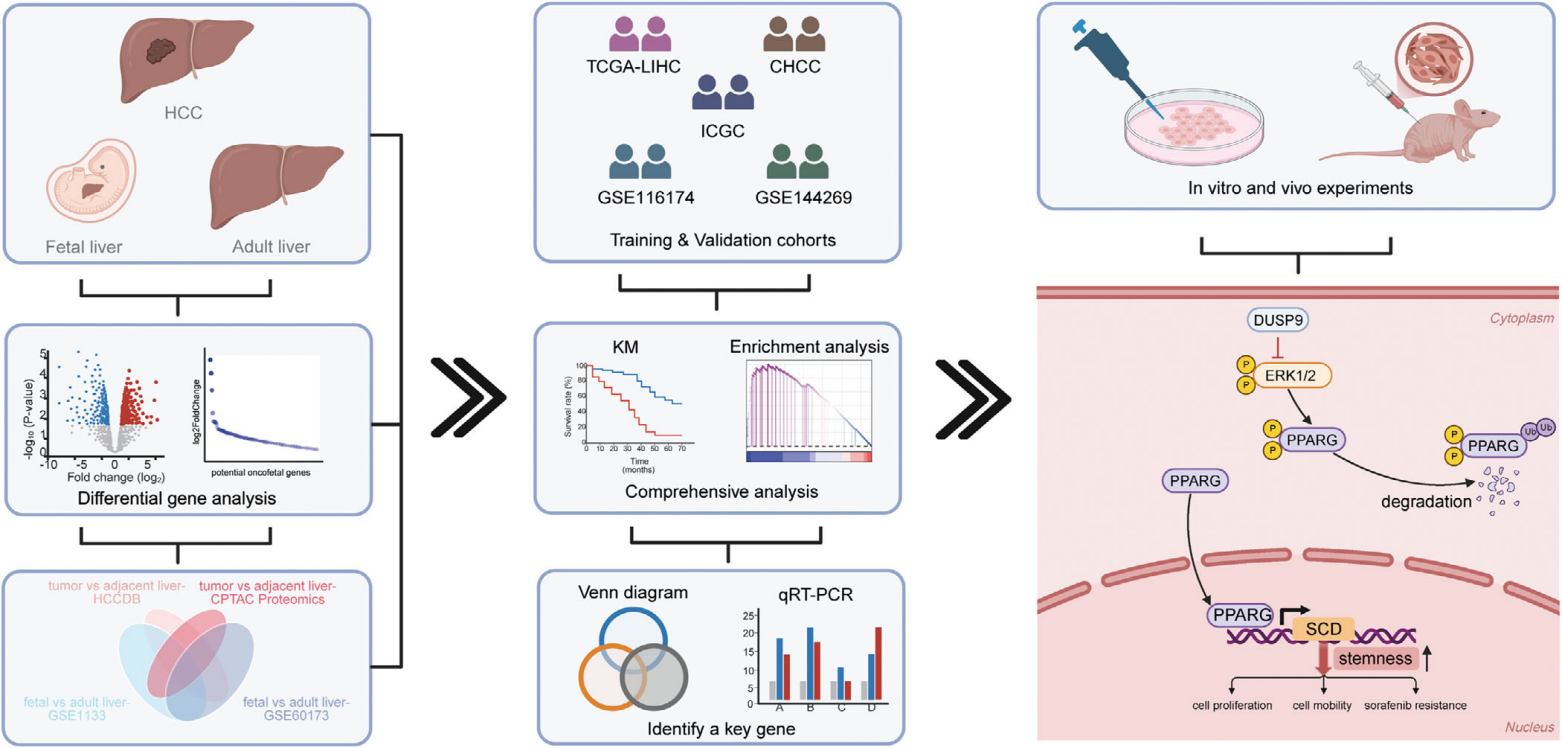
Schematic diagram of this study.

Together, these results reveal a mechanistic cascade wherein DUSP9 sustains PPARG protein stability by inhibiting ERK1/2 phosphorylation, thereby facilitating PPARG‐mediated transcriptional activation of SCD. This DUSP9–ERK1/2–PPARG–SCD axis regulates lipid metabolism and enhances stemness in HCC (Figure 10).

## 4 DISCUSSION

Despite certain advances in the prevention and treatment of HCC, the rising incidence and persistently poor clinical outcomes continue to pose a major global health challenge, underscoring critical gaps in our understanding of HCC pathogenesis.^7^ A deeper insight into the molecular mechanisms driving HCC initiation and progression is therefore urgently needed. Oncofetal proteins, exhibiting similar expression patterns in tumour cells and embryonic stem cells, have attracted attention as potential biomarkers and therapeutic targets due to their restricted expression in normal adult tissues.^32^, ^33^ In this study, we initially explored the landscape of oncofetal reprogramming in HCC using multi‐omics data and identified 23 key oncofetal genes, which were then used to construct an oncofetal reprogramming‐based prognostic signature (oncoScore). The oncoScore demonstrated robust prognostic value across five HCC cohorts and oncoScore‐high tumours were characterized by stemness‐related pathways and aggressive clinical features oncoScore‐high tumours were characterized by stemness‐related pathways and aggressive clinical features. Among these 23 genes, we further identified DUSP9 as a novel oncofetal protein associated with poor prognosis in HCC. Through a combination of in vitro and in vivo experiments, we demonstrated that DUSP9 overexpression promotes HCC stemness and progression.

Several oncofetal proteins have previously been described in HCC, with AFP being the most widely used biomarker in clinical settings.^34^ However, the biological function of AFP in HCC remains poorly understood. Other candidates such as SALL4, GPC3 and HLF have been proposed as functional oncofetal regulators, yet their precise roles in HCC pathogenesis are still under investigation.^7^ Given the profound heterogeneity of HCC driven by diverse aetiologies and complex molecular alterations, which impedes precise eradication of malignant cells and substantially limits therapeutic efficacy, there is a pressing need for unbiased approaches to identify robust, functionally relevant targets.^35^ To address this, we used large‐scale multi‐omics datasets and identified DUSP9 as a key oncofetal candidate. *DUSP9* was found to be highly expressed in fetal liver, HCC tissues and HCC‐derived organoids but minimally expressed in adult liver, adjacent tissues and healthy liver organoids. Furthermore, *DUSP9* expression was positively correlated and co‐expressed with known oncofetal genes and HCC stemness markers at both bulk and single‐cell resolution. Our tissue microarray analysis further revealed that high DUSP9 expression was significantly associated with poor OS, highlighting its prognostic value in HCC.

DUSP9 has been implicated in the tumourigenesis and progression of various malignancies.^36^, ^37^ In HCC, emerging evidence has suggested its involvement in cellular proliferation,^38^ but its broader functional roles and underlying mechanisms remain largely unexplored. Notably, CSCs share molecular and phenotypic similarities with embryonic stem cells. Building on our initial observation that *DUSP9* positively correlates with stemness‐associated genes, we conducted mechanistic investigations and demonstrated that DUSP9 overexpression enhanced self‐renewal, proliferation, migration and invasion in HCC cells. It also increased resistance to sorafenib, the standard‐of‐care therapy for advanced HCC. Using limiting dilution tumourigenicity assays, we showed that DUSP9 knockdown significantly increased the minimal cell number required for tumour initiation in vivo. These findings establish DUSP9 as a critical driver of HCC stemness and tumour‐initiating capacity.

Metabolic reprogramming is a hallmark of cancer and a prerequisite for sustaining rapid proliferation and survival under stressful microenvironmental conditions.^11^, ^39^ CSCs are particularly reliant on dynamic metabolic adaptations to maintain their stem‐like features. Lipid metabolism, in particular, plays pivotal roles in regulating CSC plasticity and fate decisions.^13^, ^40^ For instance, lncRNA *ROPM* promotes phospholipid metabolism via PLA2G16, enhancing breast cancer stemness.^41^ Utilizing single‐cell transcriptomic analysis, we discovered significant enrichment of fatty acid metabolism pathways in *DUSP9*‐high tumour subpopulations. Consistently, DUSP9 overexpression elevated intracellular triglyceride levels and lipid droplet accumulation, as confirmed by biochemical quantification and Oil Red O staining. Notably, DUSP9 significantly upregulated SCD—a key lipogenic enzyme and known metabolic vulnerability in multiple cancers, including breast and ovarian tumors.^15^, ^42^ Recent research revealed that SCD promoted the stemness of gastric CSCs through the SQLE/cholesterol/mTOR signalling pathway.^16^ Our sequential overexpression and knockdown experiments confirmed that SCD‐mediated lipid metabolism was essential for the pro‐stemness effects of DUSP9 in HCC, reinforcing the functional significance of the DUSP9–SCD axis.

Mechanistically, DUSP9 is a dual‐specificity phosphatase that inactivates MAPK/ERK signalling by dephosphorylating both threonine/serine and tyrosine residues.^27^ Given its transcriptional regulation of multiple lipid metabolism enzymes, we hypothesized that the DUSP9‐ERK1/2 axis promoted lipid metabolism by activating key transcriptional regulators. Indeed, pathway enrichment analysis revealed significant activation of PPAR signalling in *DUSP9*‐positive cells. PPARG, a master regulator of adipogenesis and lipid metabolism,^43^ has been implicated in HCC development, sorafenib resistance and metastasis.^29^, ^44^, ^45^ Prior studies have shown that phosphorylation of PPARG by ERK1/2 facilitates its ubiquitination and proteasomal degradation.^30^, ^46^ In our study, DUSP9 overexpression led to decreased ERK1/2 phosphorylation and increased PPARG protein levels, whereas DUSP9 knockdown had the opposite effect. Co‐IP confirmed interactions among DUSP9, ERK1/2 and PPARG. These results support a model in which DUSP9 stabilizes PPARG by dephosphorylating ERK1/2. As a direct transcriptional activator of SCD, increased PPARG subsequently upregulates SCD expression, promoting lipid metabolism and stemness.

Sorafenib, a multi‐kinase inhibitor, remains a frontline systemic therapy for advanced HCC. However, therapeutic resistance develops rapidly, with clinical benefit observed in only a minority of patients.^47^ Identifying molecular determinants of sorafenib sensitivity is thus of high clinical relevance. Our findings demonstrate that DUSP9 enhances resistance to sorafenib via SCD‐dependent lipid metabolism, suggesting that the DUSP9–SCD axis may dictate treatment response in a subset of HCC patients. Therapeutically, targeting this axis could sensitize tumours to sorafenib and potentially improve clinical outcomes.

Several limitations of our study should be acknowledged. First, although our single‐cell analysis provides complementary evidence to our comprehensive bulk tissue analyses, validation in larger single‐cell cohorts would further strengthen these findings. Second, our prognostic benchmarking did not include contemporary multi‐parameter serologic scores such as GALAD or ASAP, which integrate demographic variables and multiple tumour markers and have been reported to outperform AFP alone for HCC detection and risk stratification. Future external validation in prospective cohorts with complete biomarker panels will be important to enable direct comparison with, and potential integration into, established composite scores.

Although the conceptual importance of oncofetal reprogramming in tumour progression has been established, and individual studies have reported roles of specific oncofetal proteins in HCC development, our work significantly extends this field in several key aspects. First, previous studies largely focused on individual proteins with oncofetal expression characteristics, with some studies selecting target molecules merely based on their reported oncofetal features in other cancer types rather than oncofetal patterns in HCC. This study employed a multi‐omics approach integrating fetal‐adult liver transcriptomics, HCC transcriptomics and HCC proteomics to systematically identify oncofetal genes with authentic oncofetal expression patterns specifically in HCC. We further provide the characterization of 23 oncofetal proteins in HCC and demonstrate their pan‐cancer relevance, establishing their broad research value beyond individual case studies. Second, we developed and validated the oncofetal signature (oncoScore) with robust prognostic performance across HCC cohorts. Importantly, we also compared oncoScore with established oncofetal biomarkers reported in the literature, demonstrating that our signature provides superior prognostic value compared to those reported oncofetal markers. Third, we validated the DUSP9‐ERK1/2‐PPARG‐SCD axis linking oncofetal reprogramming to lipid metabolism and stem‐like properties. Our findings thus extend the role of oncofetal reprogramming in HCC prognosis and progression and suggest that targeting the DUSP9–SCD axis may represent a promising strategy for HCC treatment.

## AUTHOR CONTRIBUTIONS

**Wang Peng**: Conceptualization; in vitro experiments; in vivo experiments; bioinformatics analysis; data curation and analysis; original draft writing. **Hai Huang**: In vivo experiments. **Yuchong Zhao**: Clinical specimen collection; supervision. **Qiaodan Zhou**: Bioinformatics analysis. **Mengdie Cao**: Data curation and analysis. **Luyao Liu**: Data curation and analysis. **Jingwen Liang**: Data curation and analysis. **Haochen Cui**: Draft revision. **Shiru Chen**: Draft revision. **Wei Chen**: Clinical specimen collection. **Si Xiong**: Clinical specimen collection. **Bin Cheng**: Conceptualization; supervision. **Shuya Bai**: Conceptualization; in vivo experiments; clinical specimen collection; original draft writing; supervision. All authors reviewed the manuscript.

## CONFLICT OF INTEREST STATEMENT

The authors declare no conflicts of interest.

## ETHICS STATEMENT

All work related to human tissues was approved by the ethics committee of Tongji Hospital, HUST, Wuhan, China (IRB ID: TJIRB20230234). Informed consent was obtained from all patients for being included in the study. All animal experiments were performed in accordance with the Tongji Hospital Institutional Review Board (IRB ID: TJH‐202212058).

## Supporting information



Supporting Information

## Data Availability

Datasets from the TCGA, GEO, NODE, DepMap, LIMORE, HCCDB and CPTAC databases are publicly available. All datasets were downloaded directly from the indicated websites (Table S1).
