# Supplementary material for: Oncofetal dual‑specificity phosphatase 9 drives stem‐like properties through ERK1/2‐PPARG‐SCD axis‐mediated lipid metabolism in hepatocellular carcinoma
Source: Clin Transl Med. 2025 Dec 12;15(12):e70550. doi: 10.1002/ctm2.70550 (PMC12699142; doi:10.1002/ctm2.70550)
Supplement: Supplementary file 1 — Supporting Information [file CTM2-15-e70550-s001.pdf]

Supplementary Figures and Legends:

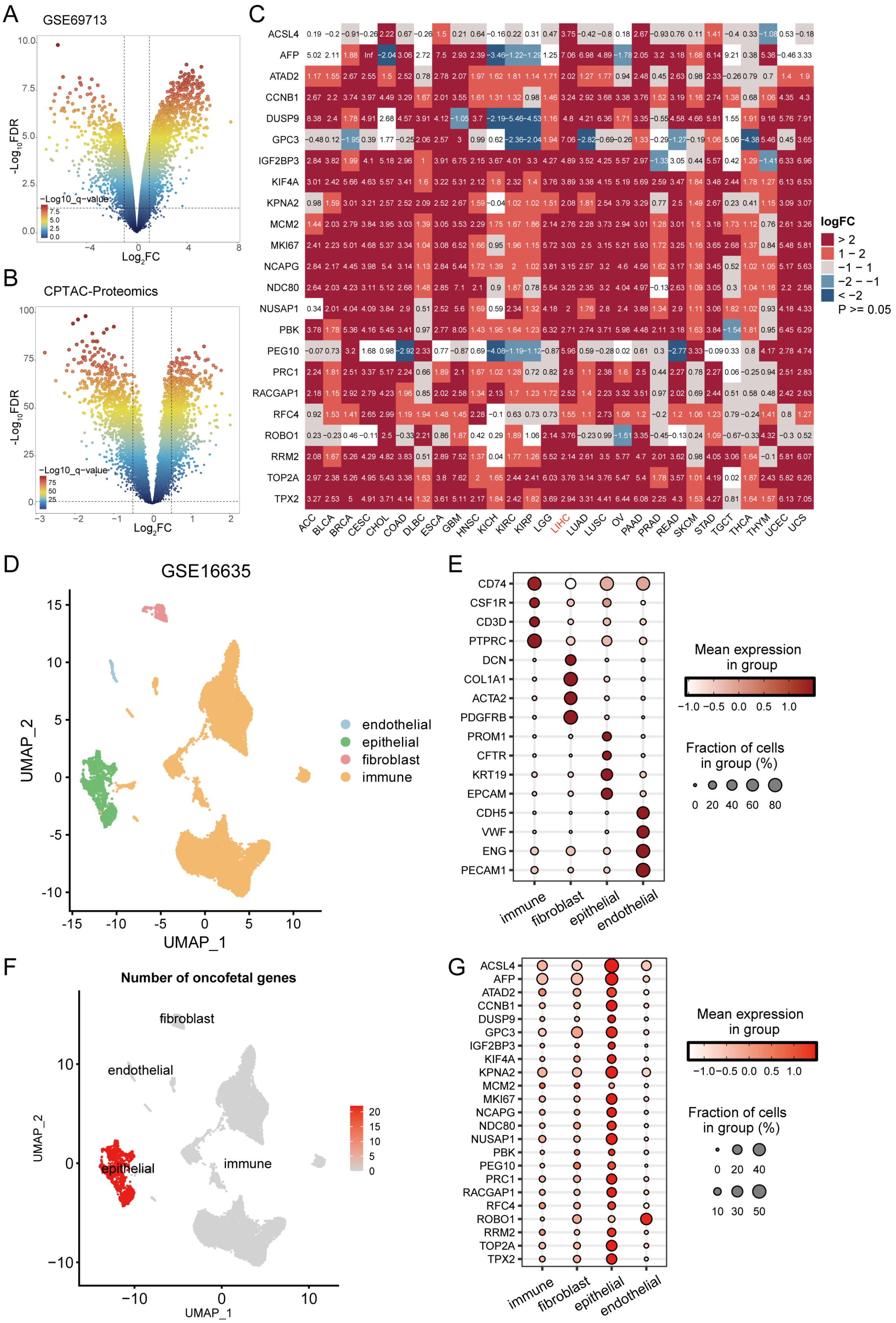

Figure S1. Identified oncofetal genes are elevated in tumors and HCC cells. (A) Volcano plot showing differentially expressed genes in the GSE69713 dataset (fetal vs. adult liver). **(B)** Volcano plot

showing differentially expressed proteins in the CPTAC dataset (tumor vs. adjacent liver). **(C)** Twenty-three oncofetal genes identified in HCC are mostly upregulated in pan-cancer tumor tissues. **(D)** UMAP plot showing four main cell types in HCC single-cell RNA-seq data (GSE16635). **(E)** Dot plot showing cell markers used for cell type annotation. **(F)** UMAP plot showing the number of highly expressed oncofetal genes in four main cell types in HCC single-cell RNA-seq data. (**G**) Dot plot showing expression levels of twenty-three oncofetal genes.

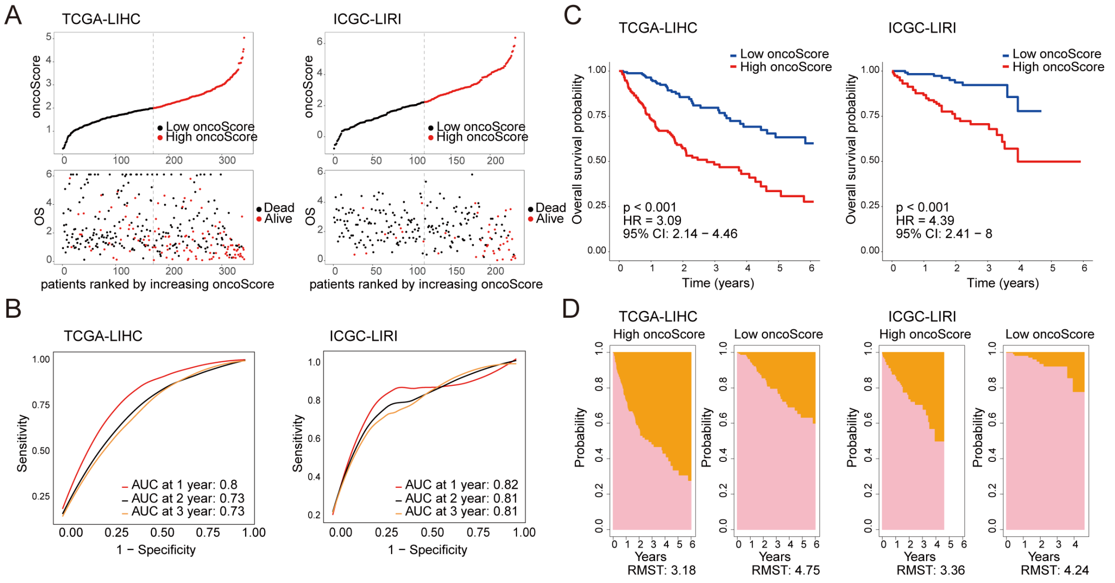

Figure S2. Validation of oncofetal reprogramming-based prognostic signature in HCC patients across public datasets. (**A**) Distribution of the oncoScore signature and overall survival status of HCC patiens in the TCGA-LIHC, and ICGC-LIRI datasets. **(B)** Time-dependent ROC curves for 1-year, 2-year, and 3-year OS across HCC datasets. **(C)** Kaplan-Meier survival analysis of OS for patients with high- and low-oncoScore across HCC datasets. **(D)** Restricted mean survival time between patients with high- and low-oncoScore across HCC datasets. Survival analysis was performed using the log-rank test. AUC, area under the Receiver Operating Characteristic curve; HR, hazard ratio; CI, confidence interval; RMST, restricted mean survival time.

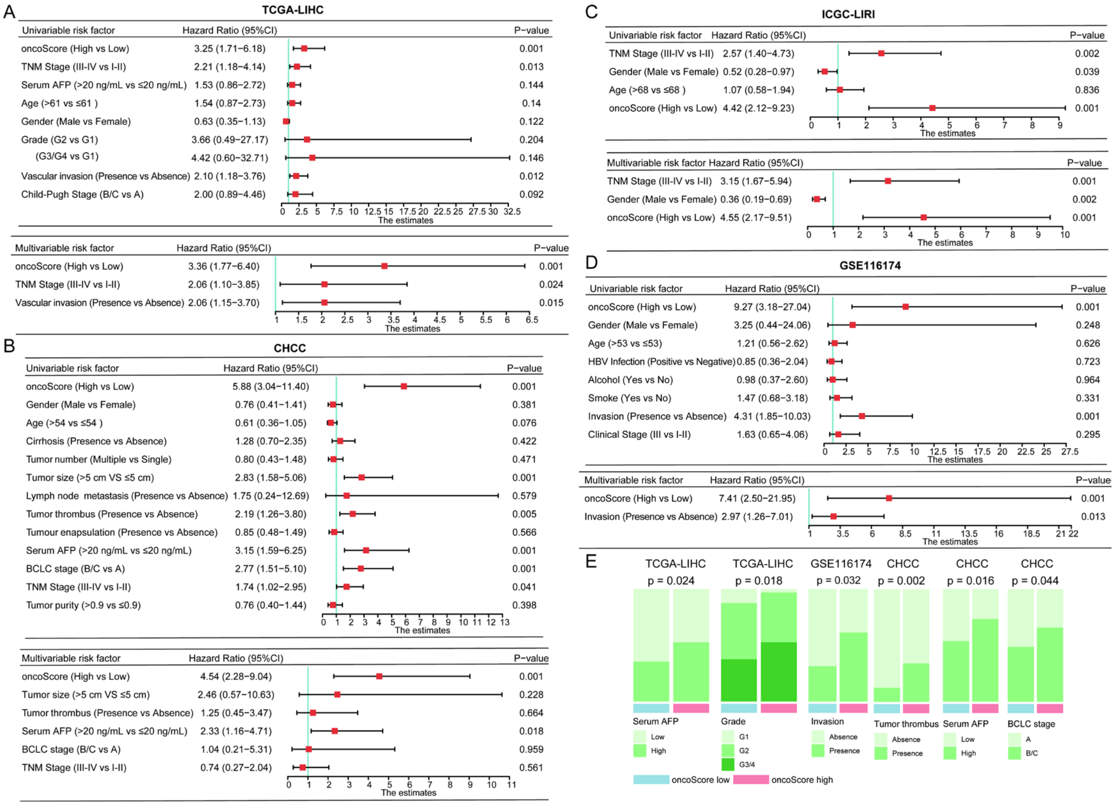

Figure S3. The oncoScore signature predicts poor prognosis in HCC patients. (A) Forest plot showing univariate and multivariate Cox regression analysis of risk factors for the overall survival in the TCGA-LIHC dataset. **(B)** Forest plot showing univariate and multivariate Cox regression analysis of risk factors for the overall survival in the CHCC dataset. **(C)** Forest plot showing univariate and multivariate Cox regression analysis of risk factors for the overall survival in the ICGC-LIRI dataset. **(D)** Forest plot showing univariate and multivariate Cox regression analysis of risk factors for the overall survival in the GSE116174 dataset. **(E)** Association between the oncoScore and clinical characteristics (serum AFP levels, tumor grade, invasion status, tumor thrombus, and BCLC stage) in public HCC datasets. Chi-square test and Fisher's exact test was used to analyze the correlation between categorical variables. CI, confidence interval.

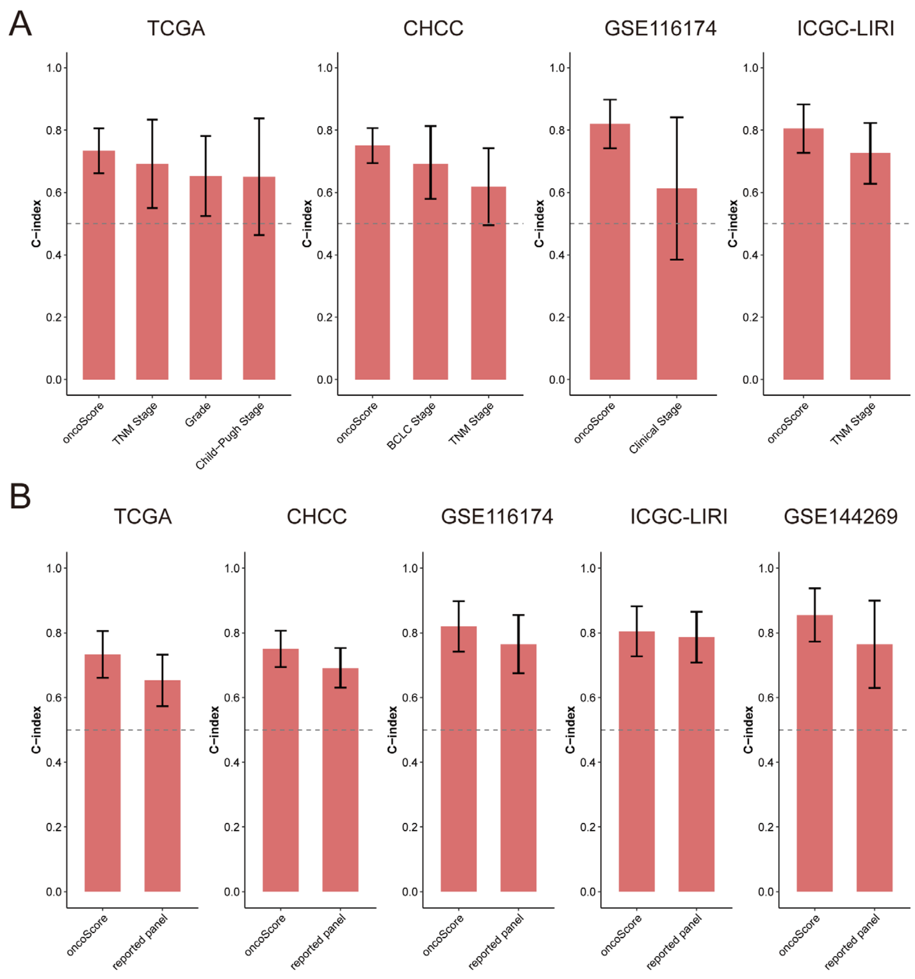

Figure S4. The C-index of oncoScore, clinical staging systems, and reported oncofetal biomarker panel. (**A**) C-index plots of oncoScore and clinical staging systems across HCC datasets. (**B**) C-index plots of oncoScore and reported oncofetal biomarker panel across HCC datasets.

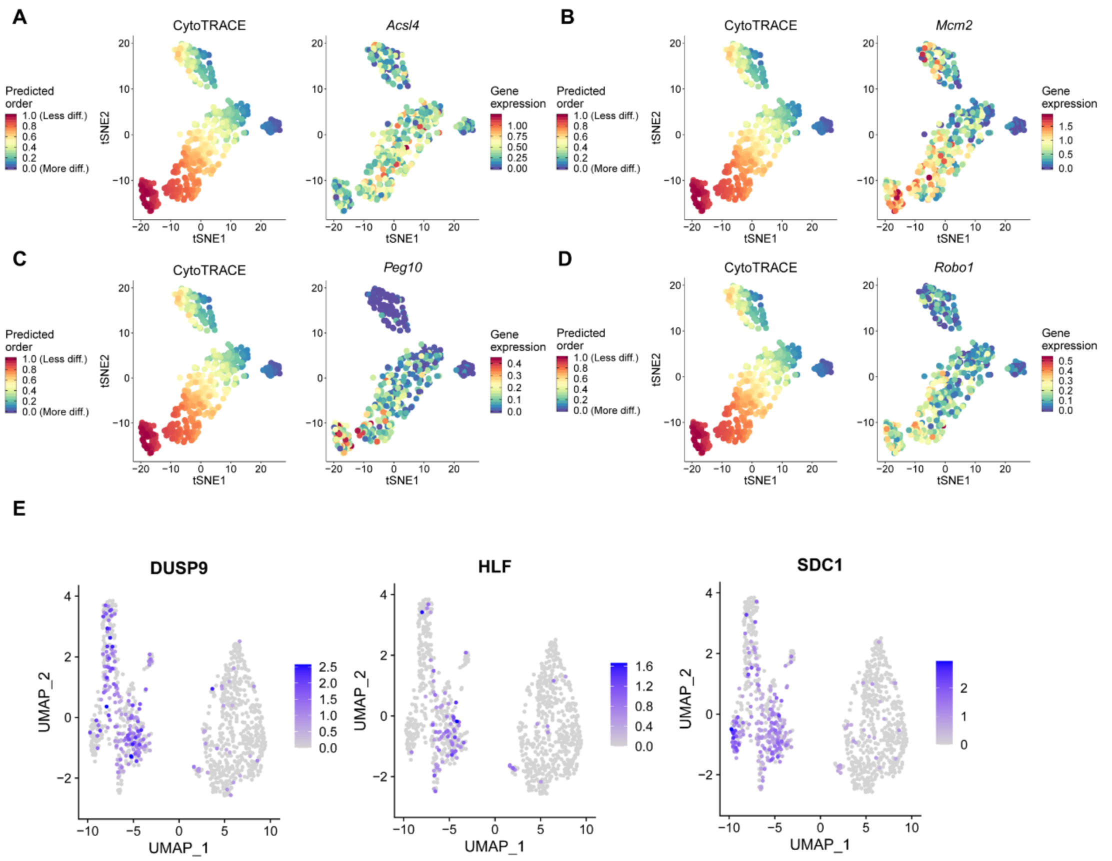

Figure S5. DUSP9 is co-expressed with oncofetal and stemness-associated genes at the single-cell level. (**A-D**) CytoTRACE pseudotime analysis of hepatoblast single-cell sequencing data and expression levels of Acsl4 **(A)**, Mcm2 **(B)**, Peg10 **(C)**, and Robo1 **(D)**. Left: cell differentiation states (red indicates lower differentiation). Right: gene expression levels (red indicates higher expression). (**E**) Expression levels of DUSP9, HLF, and SDC1 in single-cell HCC subclusters.

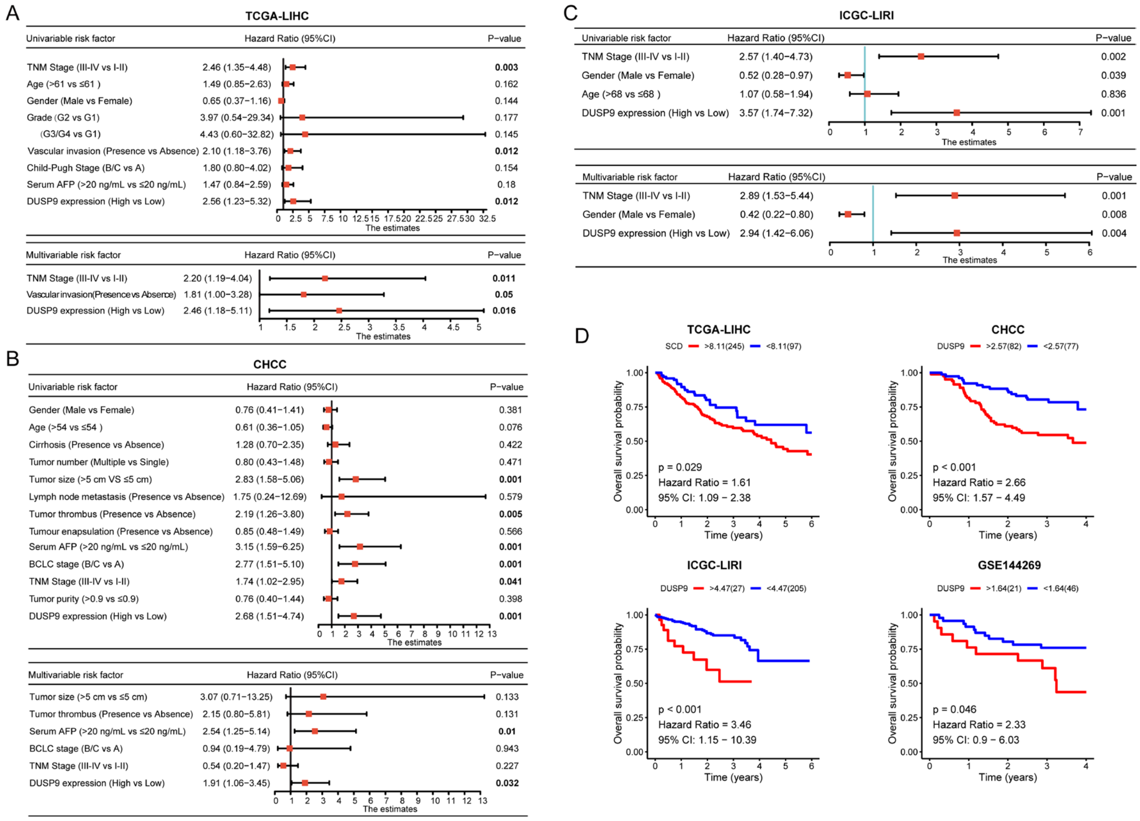

Figure S6. DUSP9 expression correlates with poor prognosis in HCC patients. (A) Forest plot showing univariate and multivariate Cox regression analysis of risk factors for the overall survival in the TCGA-LIHC dataset. **(B)** Forest plot showing univariate and multivariate Cox regression analysis of risk factors for the overall survival in the CHCC dataset. **(C)** Forest plot showing univariate and multivariate Cox regression analysis of risk factors for the overall survival in the ICGC-LIRI dataset. **(D)** Kaplan-Meier survival curves for patients with high vs. low DUSP9 expression in the TCGA-LIHC, CHCC, ICGC-LIRI, and GSE144269 datasets. Survival analysis was performed using the log-rank test. CI, confidence interval.

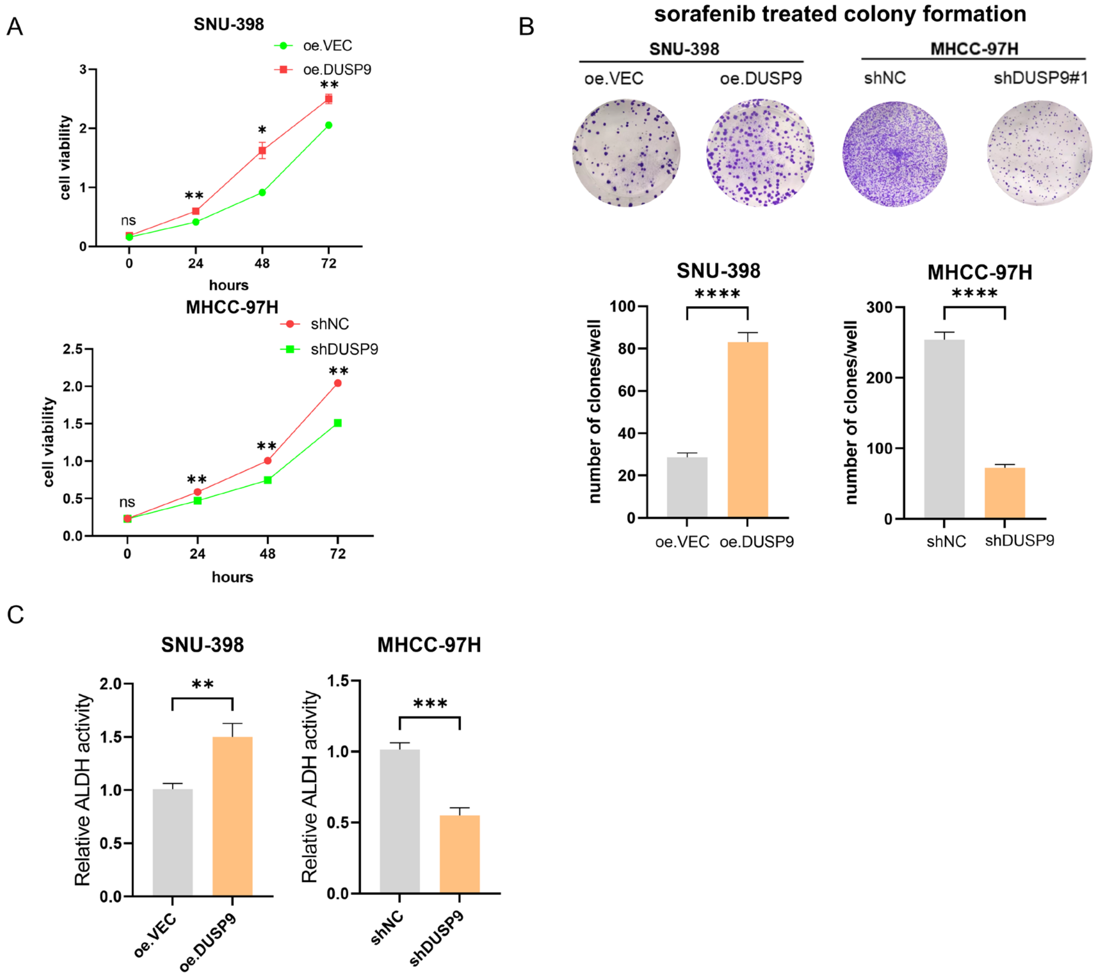

Figure S7. DUSP9 promotes stem-like properties of HCC cells. (A) CCK8 proliferation assays showing cell proliferation abilities in DUSP9-modulated cells. **(B)** Sorafeinb treated long-term cloning formation assays showing sorafenib resistance abilities in DUSP9-modulated cells. **(C)** ALDH activity assays showing ALDH activities in DUSP9-modulated cells. The results are presented as mean ± SD, and analyzed using Student's t-test. ns $P > 0.05$; * $P < 0.05$; ** $P < 0.01$.

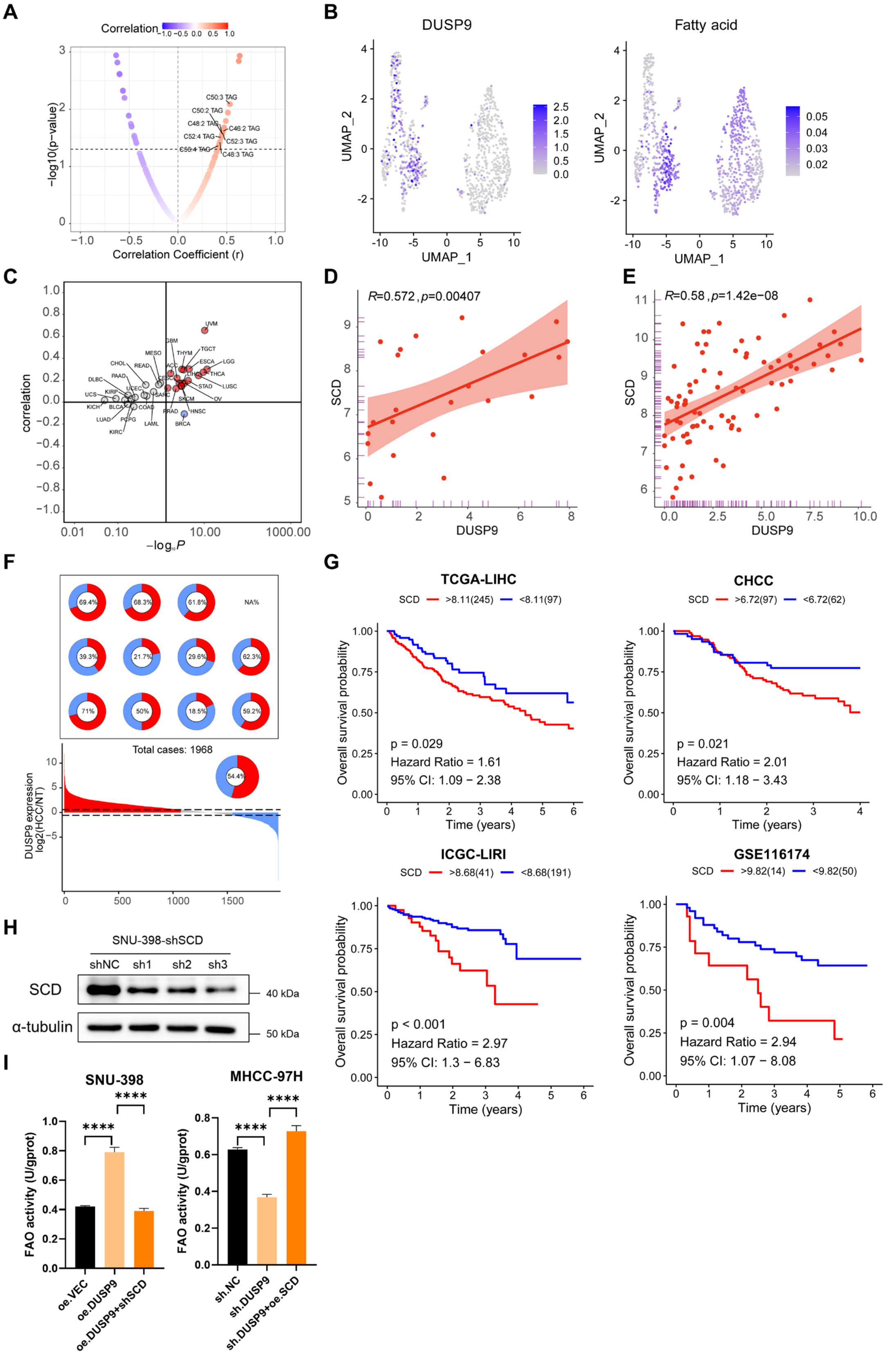

Figure S8. SCD mediates DUSP9-driven lipid metabolic reprogramming in HCC. (A) Volcano plot showing the correlation between DUSP9 expression and metabolite abundance in HCC cell lines in Depmap database. Annotated: positively correlated triglycerides. **(B)** Expression levels of DUSP9 and

scFEA-inferred fatty acid metabolism activity across single-cell HCC subpopulations (GSE16635). (**C-E**) Correlation between DUSP9 and SCD expression in TCGA pan-cancer transcriptomic data **(C)**, Depmap 24 HCC cell lines **(D)**, and LIMORE 81 HCC cell lines **(E)**. (**F**) SCD expression profiles across 11 bulk HCC transcriptomic datasets from the HCCDB database. (**G**) Kaplan-Meier survival curves for patients with high vs. low SCD expression in the TCGA-LIHC, CHCC, ICGC-LIRI, and GSE116174 datasets. **(H)** Knockdown efficiency of SCD in SNU-398 cells. **(I)** Fatty acid oxidation (FAO) activities in DUSP9–SCD sequential-modulated HCC cells. The results are presented as mean ± SD, and analyzed using Student's t-test or one-way ANOVA followed by Tukey's post-hoc test for multiple comparisons as appropriate. Survival analysis was performed using the log-rank test. Spearman's rank correlation test was used to analyze the correlation between continuous variables. CI, confidence interval.

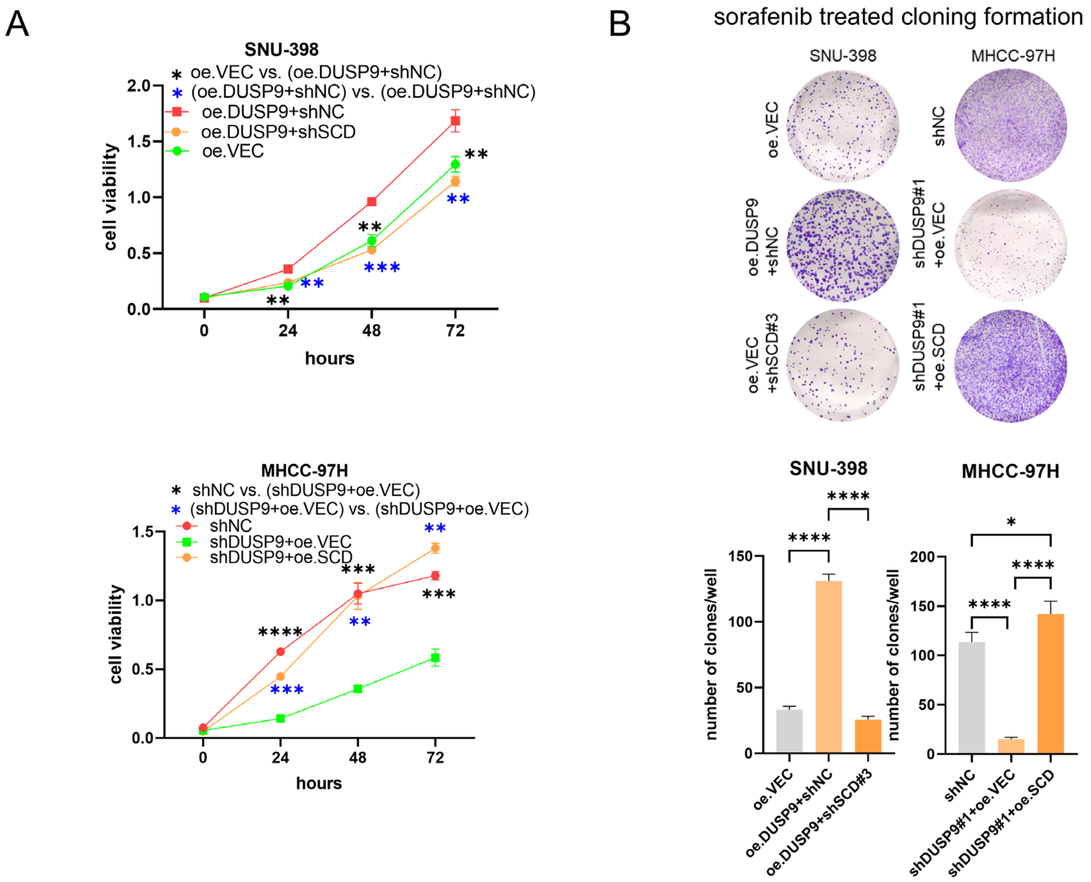

Figure S9. SCD mediates stem-like properties of DUSP9 in HCC cells. (A) CCK8 proliferation assays showing cell proliferation abilities in DUSP9–SCD sequential-modulated HCC cells. **(B)** Sorafeinb treated long-term cloning formation assays showing sorafenib resistance abilities in DUSP9–SCD sequential-modulated HCC cells. The results are presented as mean ± SD, and analyzed using Student's t-test or one-way ANOVA followed by Tukey's post-hoc test for multiple comparisons as appropriate. ** $P < 0.01$; *** $P < 0.001$; **** $P < 0.0001$.

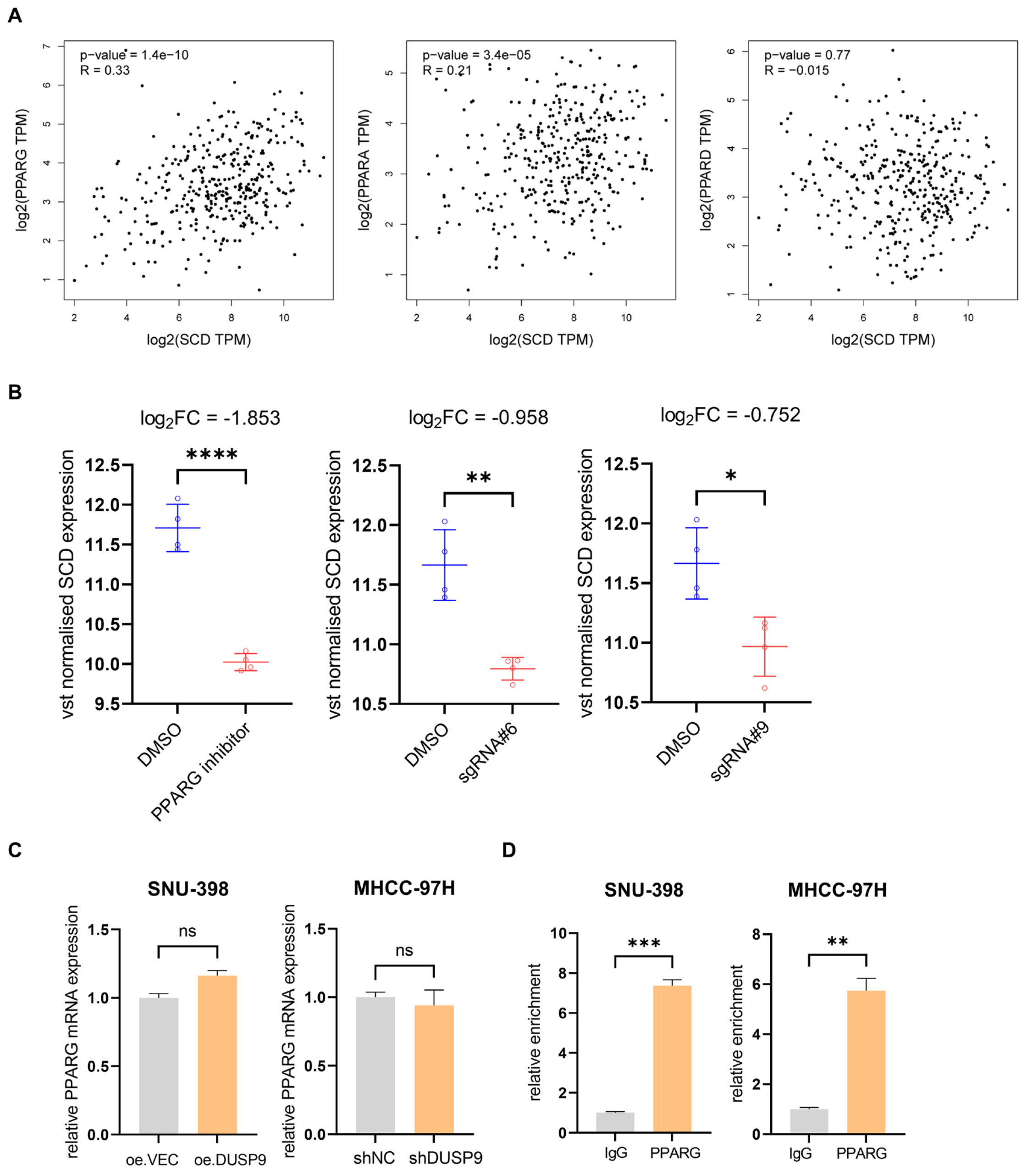

Figure S10. The relationship among DUSP9, PPARG, and SCD. (A) Scatter plot showing the correlation between SCD and PPARG/PPARA/PPARD mRNA levels in TCGA-LIHC dataset. **(B)** Relative SCD mRNA expression in PPARG-inhibited (left) and PPARG-knockout (middle and right) bladder urothelial carcinoma cells (GSE166801). **(C)** QRT-PCR analysis of PPARG mRNA expression in DUSP9-overexpressing SNU-398 cells (left) and DUSP9-knockdown MHCC-97H cells (right). The results are presented as mean ± SD; ns $P > 0.05$; * $P < 0.05$; ** $P < 0.01$; **** $P < 0.0001$**. (C)** Scatter plot showing the correlation between DUSP9 mRNA levels and ERK1/2 phosphorylation in HCC cell lines from the DepMap dataset. The results are presented as mean ± SD, and analyzed using Student's t-test. Spearman's rank correlation test was used to analyze the correlation between continuous variables.

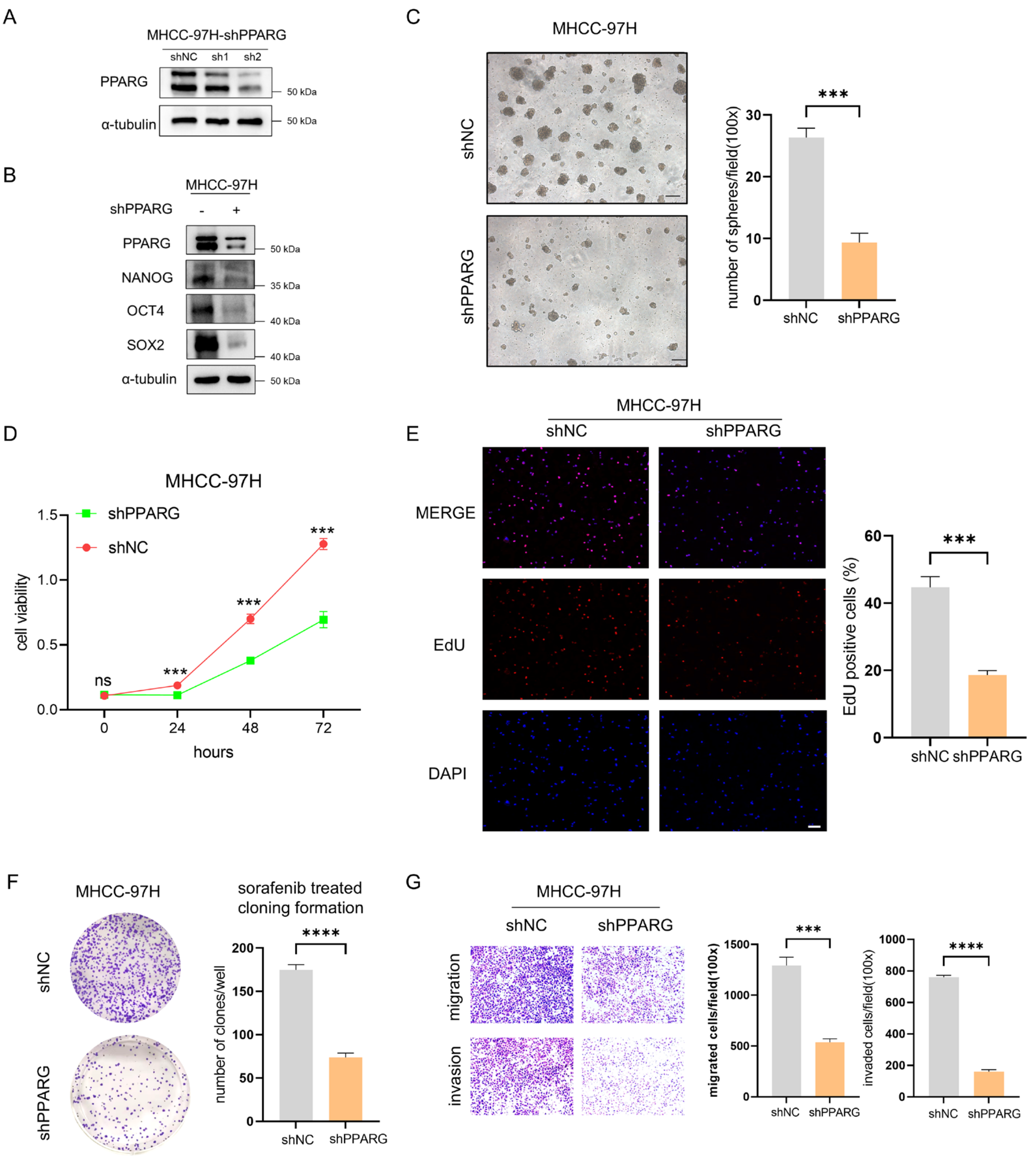

Figure S11. PPARG knockdown impairs stem-like properties of MHCC-97H cells. (A) Knockdown efficiency of PPARG in MHCC-97H cells. **(B)** Western blot of stemness genes (SOX2, OCT4, NANOG) in PPARG-knockdown MHCC-97H cells. **(C)** Sphere formation assays assessing self-renewal ability (scale bar: 100 μm). **(D)** CCK8 proliferation assays showing cell proliferation abilities in PPARG-knockdown MHCC-97H cells. **(E)** EdU proliferation assays of PPARG-knockdown MHCC-97H cells. **(F)** Sorafeinb treated long-term cloning formation assays showing sorafenib resistance abilities in PPARG-knockdown MHCC-97H cells. **(G)** Transwell migration and invasion assays of PPARG-knockdown MHCC-97H cells. The results are presented as mean ± SD, and analyzed using Student's t-test. ns $P > 0.05$; * $P < 0.05$; ** $P < 0.01$; **** $P < 0.0001$.

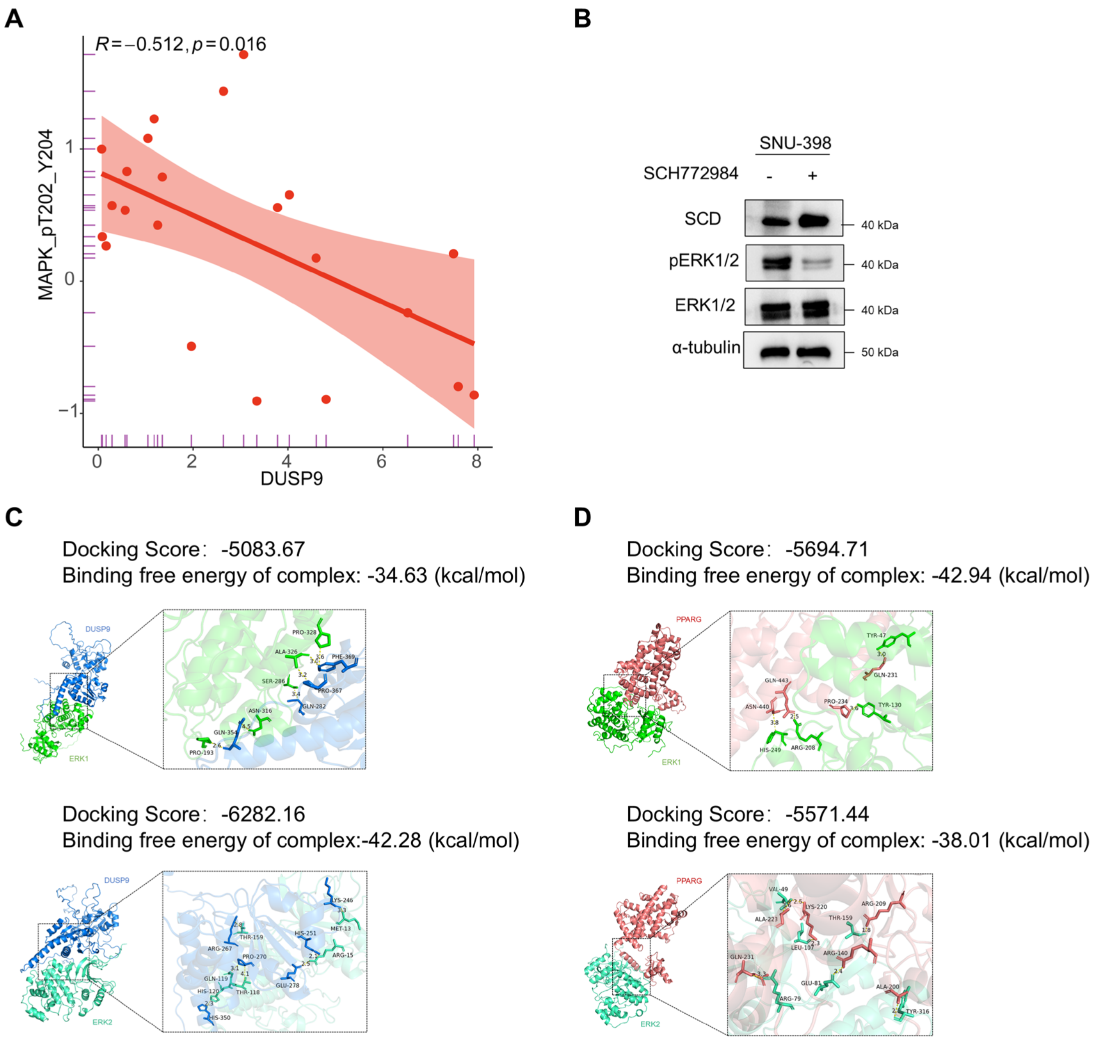

Figure S12. The relationship among DUSP9, PPARG, SCD and ERK1/2. (A) Scatter plot showing the correlation between DUSP9 mRNA levels and ERK1/2 phosphorylation in HCC cell lines from the DepMap dataset. **(B)** SCD expression in ERK1/2 inhibitor SCH772984-treated SNU-398 cells. **(C)** Molecular docking of DUSP9 and ERK1/2 proteins. **(D)** Molecular docking of PPARG and ERK1/2 proteins. Spearman's rank correlation test was used to analyze the correlation between continuous variables.

Supplementary Tables:

Table S1. Public available datasets/database used in this study.

Dataset/Database	Data type	Website
HCCDB	12 datasets of HCC bulk transcriptomic data	http://lifeome.net/database/hccdb/home
GSE69713	Human fetal liver bulk transcriptomic data	https://www.ncbi.nlm.nih.gov/geo/
GSE1133	Human fetal liver bulk transcriptomic data	https://www.ncbi.nlm.nih.gov/geo/
GSE116174	Human HCC bulk transcriptomic data	https://www.ncbi.nlm.nih.gov/geo/
GSE144269	Human HCC bulk transcriptomic data	https://www.ncbi.nlm.nih.gov/geo/
GSE84073	Bulk transcriptomic data of Liver cancer and normal liver organoid	https://www.ncbi.nlm.nih.gov/geo/
GSE166635	HCC single-cell RNA transcriptomic data	https://www.ncbi.nlm.nih.gov/geo/
CPTAC	HCC proteomic data	https://pdc.cancer.gov/pdc/
TCGA	Pan-cancer bulk transcriptomic data	https://portal.gdc.cancer.gov/
ICGC	Human HCC bulk transcriptomic data	https://icgc.org/
OEP000321	Human HCC bulk transcriptomic data	https://www.biosino.org/node/
Depmap	Pan-cancer cell line metabolomic, bulk transcriptomic, and proteomic data	https://depmap.org
Cytotrace	Hepatoblast single-cell RNA transcriptomic data	https://cytotrace.stanford.edu/
GSE166801	Bulk transcriptomic data of PPARG inhibition/ knockout in bladder urothelial carcinoma	https://www.ncbi.nlm.nih.gov/geo/
GSE97098	81 HCC cell lines bulk transcriptomic data	https://www.ncbi.nlm.nih.gov/geo/

Table S2. Primer Sequences for Quantitative Real-time PCR Analysis.

Genes	Forward Primer Sequence (5' to 3')	Reverse Primer Sequence (5' to 3')
ACTB (human)	CATGTACGTTGCTATCCAGGC	CTCCTTAATGTCACGCACGAT
DUSP9 (human)	AGAAGCTGCGAGAGGAAGGCTA	CAGCAAGGCTGGTCTCACACAG
FASN (human)	AAGGACCTGTCTAGGTTTGATGC	TGGCTTCATAGGTGACTTCCA
SCD (human)	TCTAGCTCCTATACCACCACCA	TCGTCTCCAACTTATCTCCTCC
ACLY (human)	TCGGCCAAGGCAATTTCAGAG	CGAGCATACTTGAACCGATTCT
ACACA (human)	ATGTCTGGCTTGCACCTAGTA	CCCCAAAGCGAGTAACAAATTCT
HMGCR (human)	GACGTGAACCTATGCTGGTCAG	GGTATCTGTTTCAGCCACTAAG

		G
HMGCS1 (human)	AAGTCACACAAGATGCTACACCG	TCAGCGAAGACATCTGGTGCCA
Actb (mouse)	GGCTGTATTCCCCTCCATCG	CCAGTTGGTAACAATGCCATGT
Dusp9 (mouse)	GCCAAAGAAGAGTGGGATGCTG	GTTTCACACAGGTGAGGACACTC
Acsl4 (mouse)	CCTTTGGCTCATGTGCTGGAAC	GCCATAAGTGTGGGTTTCAGTAC
Mcm2 (mouse)	CCGTTCCAAGGATGCCATTCTC	TGGAAAGCCGTTGGCGGTGTTA
Peg10 (mouse)	AGGCGGATATGCAGCACCAACT	TGCTCATGCTGATCTGGAGGCA
Robo1 (mouse)	GGCAGCCTGTTGGATTATCCTC	GTTGGCGTGAAGGTAAAGGACG

Table S3. Primer Sequences for Plasmid Construction (Overexpression and Knockdown).

Target	Regulation	Primer Name	Primer Sequence (5' to 3')
DUSP9 (human)	Overexpression	Forward	TACCGGACTCAGATCTCGAGCCACCA
		Reverse	TATCTAGATCCGGTGGATCCCTAGGTGGGGGCCAGCTCGAAGG
	Knockdown-sh1	Forward	CCGGCCTCATGCAGAAGCTCCACCTCTCGAGAGGTGGAGCTTCTGCATGAGGTTTTTG
		Reverse	AATTCAAAAACCTCATGCAGAAGCTCCACCTCTCGAGAGGTGGAGCTTCTGCATGAGG
	Knockdown-sh2	Forward	CCGGCTTCAGCAGATTCCAGGCCGACTCGAGTCGGCCTGGAATCTGCTGAAGTTTTTG
		Reverse	AATTCAAAAACTTCAGCAGATTCCAGGCCGACTCGAGTCGGCCTGGAATCTGCTGAAG
	Knockdown-sh3	Forward	CCGGGAGGATCTATGCTTGTTTGTTCTCGAGAACAAACAAGCATAGATCCTCTTTTTG
		Reverse	AATTCAAAAAGAGGATCTATGCTTGTTTGTTCTCGAGAACAAACAAGCATAGATCCTC
SCD (human)	Overexpression	Forward	CCCTCGAGGCCACCATGCCGGCCCACTTGCTG
		Reverse	CGGGATCCTCACTTATCGTCGTCATCCTTGTAATCGCCACTCTTGTAGTTTCCATCTCC
	Knockdown-sh1	Forward	CCGGCTACGGCTCTTTCTGATCAT

			TCTCGAGAATGATCAGAAAGAGCCGTAGTTTTTG
		Reverse	AATTCAAAAACTACGGCTCTTTCTGATCATTCTCGAGAATGATCAGAAAGAGCCGTAG
	Knockdown-sh2	Forward	CCGGCGTCCTTATGACAAGAACATTCTCGAGAATGTTCTTGTCATAAGGACGTTTTTG
		Reverse	AATTCAAAAACGTCCTTATGACAAGAACATTCTCGAGAATGTTCTTGTCATAAGGACG
	Knockdown-sh3	Forward	CCGGCCCACCTACAAGGATAAGGAACTCGAGTTCCTTATCCTTGTAGGTGGGTTTTTG
		Reverse	AATTCAAAAACCCACCTACAAGGATAAGGAACTCGAGTTCCTTATCCTTGTAGGTGGG
PPARG (human)	Knockdown-sh1	Forward	CCGGCAGCATTTCTACTCCACATTACTCGAGTAATGTGGAGTAGAAATGCTGTTTTTG
		Reverse	AATTCAAAAACAGCATTTCTACTCCACATTACTCGAGTAATGTGGAGTAGAAATGCTG
	Knockdown-sh2	Forward	CCGGATGGAGTCCACGAGATCATTTCTCGAGAAATGATCTCGTGGACTCCATTTTTG
		Reverse	AATTCAAAAAATGGAGTCCACGAGATCATTTCTCGAGAAATGATCTCGTGGACTCCAT

Table S4. List of 23 Oncofetal Genes Used in oncoScore Signature.

ACSL4	AFP	ATAD2	CCNB1	DUSP9	GPC3
IGF2BP3	KIF4A	KPNA2	MCM2	MKI67	NCAPG
NDC80	NUSAP1	PBK	PEG10	PRC1	RACGAP1
RFC4	ROBO1	RRM2	TOP2A	TPX2	

Supplementary Methods

Immunohistochemistry (IHC)

IHC staining was conducted to assess the protein expression in paired HCC/adjacent tissues. Briefly, tissue sections underwent dewaxing, rehydration, and antigen retrieval in 0.01 M citrate buffer (pH 6.0). Endogenous peroxidases were then blocked by treating the sections with 3% hydrogen peroxide for 15 minutes in the dark, followed by three PBS washes. After permeabilization with 0.5% Triton X-100 and blocking with 5% BSA, the sections were incubated with the primary antibody for 1 hour at room temperature, and subsequently with a secondary antibody for 30 minutes. Following PBS rinses, color development was initiated with DAB substrate and stopped by tap water rinsing once the desired stain intensity was achieved. Finally, the sections were counterstained with hematoxylin, dehydrated via a graded ethanol series, cleared in xylene, and imaged under a microscope.

Colony formation assay

500 cells were inoculated into 6-well plates. The medium was changed every four days. After approximately 14 days, when visible cell clones had formed, the cells were washed with PBS, fixed with 4% paraformaldehyde (PFA) for 20 minutes at room temperature, and then stained with a 0.1% crystal violet solution for 20 minutes. After three PBS washes and air-drying, the colonies were photographed for subsequent statistical analysis.

EdU Proliferation Assay

Cell proliferation was evaluated with the BeyoClick™ EdU Cell Proliferation Kit (Alexa Fluor 594; Beyotime, C0071S). Adherent cells were pulsed with 10 μM EdU for 2 hours to label replicating DNA. After PBS rinses, cells were incubated with the Click reaction cocktail (prepared according to the manufacturer's instructions) for 30 minutes in the dark. Nuclei were then counterstained with DAPI for 5 minutes. Following final washes, images were acquired using a fluorescence microscope.

Transwell migration and invasion assays

Cell migration and invasion capacities were assessed using Transwell chambers (Millipore). For migration, 5×10^4 cells in serum-free medium were seeded into the upper chamber, while the lower chamber was filled with 800 μL of DMEM containing 10% FBS to serve as a chemoattractant. Following a 24- or 48-hour incubation, non-migrated cells on the upper surface were removed with a cotton swab. The cells that had traversed the membrane were fixed with 4% PFA, stained with 0.1% crystal violet, and imaged. The invasion assay followed an identical protocol, with the exception that the upper chambers were pre-coated with a thin layer of Matrigel (BD Biosciences, 1:7 dilution). Migrated and invaded cells from five random microscopic fields per well were quantified, and all experiments were conducted in triplicate.

Triglyceride quantification assay

Cells were lysed on ice for 30 minutes with freshly prepared lysis buffer. The triglyceride detection was

followed by the instructions. 250 μL working solution and 2.5 of ddH_2O were added to the blank hole, 250 μL working solution and 2.5 μL calibration sample were added to the calibration hole, and 250 μL working solution and 2.5 μL cell lysate were added to the sample hole. After mixing, the plates were incubated at 37 °C for 10 minutes. The absorbance at 510 nm was measured using a microplate reader. The formula to calculate triglyceride content is：

$$\frac{(sample\ absorbance - blank\ absorbance) * calibrator\ concentration}{(calibrator\ absorbance - blank\ absorbance) * sample\ protein\ level}$$

Oil Red O staining

Oil Red O working solution was prepared by mixing the stock solution with ddH_2O at a 3:2 ratio, followed by filtration through a 0.22 μm filter. Cells were fixed with 4% PFA for 30 minutes, briefly treated with 60% isopropanol, and then stained with the working solution for 30 minutes at 37°C in the dark. After washing, cells were counterstained with hematoxylin and imaged under a microscope.

Immunofluorescence staining

Cells grown on glass coverslips were fixed with 4% PFA for 10 minutes, permeabilized with 0.1% Triton X-100, and blocked with 5% BSA. They were then incubated overnight at 4°C with a PPARG primary antibody (Proteintech, 16643-1-AP), followed by a fluorescent secondary antibody for 2 hours in the dark. Nuclei were stained with DAPI, and images were acquired using a fluorescence microscope.

Molecular docking

Molecular docking was performed to investigate the interactions among DUSP9 (Uniprot ID: Q99956), PPARG (Uniprot ID: P37231), ERK1 (Uniprot ID: P27361), and ERK2 (Uniprot ID: P28482). Protein structures were retrieved from the AlphaFold database (https://alphafold.ebi.ac.uk/) and prepared using PyMol 2.4. Protein-protein docking was executed on the HawkDock server (http://cadd.zju.edu.cn/hawkdock/), which employs MM/GBSA for binding free energy calculation[1, 2]. From 10 initial poses per pair, the model with the most favorable docking score and binding free energy was selected. Residue-level decomposition identified key interacting residues. The final complexes were visualized with PyMol 2.4.

References:

1. Weng, G., et al., *HawkDock: a web server to predict and analyze the protein-protein complex based on computational docking and MM/GBSA.* Nucleic Acids Res, 2019. **47**(W1): p. W322-W330.
2. Hou, T., et al., *Assessing the performance of the MM/PBSA and MM/GBSA methods. 1. The accuracy of binding free energy calculations based on molecular dynamics simulations.* J Chem Inf Model, 2011. **51**(1): p. 69-82.
